# A high‐quality *Brassica napus* genome reveals expansion of transposable elements, subgenome evolution and disease resistance

**DOI:** 10.1111/pbi.13493

**Published:** 2020-11-20

**Authors:** Xuequn Chen, Chaobo Tong, Xingtan Zhang, Aixia Song, Ming Hu, Wei Dong, Fei Chen, Youping Wang, Jinxing Tu, Shengyi Liu, Haibao Tang, Liangsheng Zhang

**Affiliations:** ^1^ Fujian Provincial Key Laboratory of Haixia Applied Plant Systems Biology Key Laboratory of Ministry of Education for Genetics & Breeding and Multiple Utilization of Crops College of Agriculture Fujian Agriculture and Forestry University Fuzhou China; ^2^ The Key Laboratory of Biology and Genetic Improvement of Oil Crops The Ministry of Agriculture and Rural Affairs of PRC Oil Crops Research Institute Chinese Academy of Agricultural Sciences Wuhan China; ^3^ College of Horticulture Nanjing Agricultural University Nanjing China; ^4^ Key Laboratory of Plant Functional Genomics of the Ministry of Education Yangzhou University Yangzhou China; ^5^ National Key Laboratory of Crop Genetic Improvement National Center of Rapeseed Improvement Huazhong Agricultural University Wuhan China; ^6^ Genomics and Genetic Engineering Laboratory of Ornamental Plants College of Agriculture and Biotechnology Zhejiang University Hangzhou China

**Keywords:** *Brassica napus*, long‐read sequencing, selective sweep, subgenome evolution, disease resistance

## Abstract

Rapeseed (*Brassica napus L*.) is a recent allotetraploid crop, which is well known for its high oil production. Here, we report a high‐quality genome assembly of a typical semi‐winter rapeseed cultivar, 'Zhongshuang11' (hereafter 'ZS11'), using a combination of single‐molecule sequencing and chromosome conformation capture (Hi‐C) techniques. Most of the high‐confidence sequences (93.1%) were anchored to the individual chromosomes with a total of 19 centromeres identified, matching the exact chromosome count of *B. napus*. The repeat sequences in the A and C subgenomes in *B. napus* expanded significantly from 500 000 years ago, especially over the last 100 000 years. These young and recently amplified LTR‐RTs showed dispersed chromosomal distribution but significantly preferentially clustered into centromeric regions. We exhaustively annotated the nucleotide‐binding leucine‐rich repeat (NLR) gene repertoire, yielding a total of 597 NLR genes in *B. napus* genome and 17.4% of which are paired (head‐to‐head arrangement). Based on the resequencing data of 991 *B. napus* accessions, we have identified 18 759 245 single nucleotide polymorphisms (SNPs) and detected a large number of genomic regions under selective sweep among the three major ecotype groups (winter, semi‐winter and spring) in *B. napus*. We found 49 NLR genes and five NLR gene pairs colocated in selective sweep regions with different ecotypes, suggesting a rapid diversification of NLR genes during the domestication of *B. napus*. The high quality of our *B. napus* 'ZS11' genome assembly could serve as an important resource for the study of rapeseed genomics and reveal the genetic variations associated with important agronomic traits.

## Introduction

Many important crops are recent allopolyploids with different sets of subgenomes that were derived from the interspecific hybridization between related species (Cheng *et al*., 2018; Zhang *et al*., 2019a). However, it is often a formidable task to obtain a high‐quality polyploid genome assembly due to the large genome size and highly similar homeologous subgenomes that tend to create much increased complexities in assembly graphs. In particular, the repetitive and complex regions present a challenge as next‐generation sequencing (NGS) or second‐generation sequencing platforms generally produce short reads that are incapable of spanning and resolving repetitive regions. In the last few years, several new technologies have become available to drastically improve existing reference genomes (Wang *et al*., 2019b; Zhang *et al*., 2020), for instance long‐read sequencing including single‐molecule real‐time (SMRT) sequencing and Oxford Nanopore, and chromosome conformation capture (Hi‐C). Based on these technologies, chromosome‐level assemblies of allopolyploid genomes were achieved. For example, the high‐quality, chromosomal‐scale reference genome of quinoa (*Chenopodium quinoa Willd*., 2*n* = 4*x* = 36) was successfully produced using the SMRT sequencing coupled with BioNano, Hi‐C and genetic maps (Jarvis *et al*., 2017). With the further development of sequencing technologies, it is likely that the quality of polyploid genomes could be much improved compared with the earlier draft genomes, providing richer information for the genetic diversity and molecular breeding of economic crops.

Allotetraploid oilseed rape (*B. napus L*., AACC, 2*n* = 38) is a member of Brassicaceae family and was thought to be derived ~7500 years ago with the hybridization of two diploid parental genomes *Brassica rapa* (AA, 2*n* = 20) (Wang *et al*., 2011) and *Brassica oleracea* (CC, 2*n* = 18) (Liu *et al*., 2014) and subsequent genome doubling (Chalhoub *et al*., 2014). There are three ecotype groups of *B. napus*, including spring, winter and semi‐winter that are adapted to different geographical environments (Lu *et al*., 2019). The widespread cultivation of rapeseed crop increased the exposure to disease caused by various pathogens, thus leading to a serious decline in yield, including blackleg (*Leptosphaeria maculans* and *L. biglobosa*), clubroot (*Plasmodiophora brassicae*) and *Sclerotinia* stem rot (*Sclerotinia sclerotiorum*) (Neik *et al*., 2017; Sanogo *et al*., 2015; Van de Wouw *et al*., 2016; Wei *et al*., 2017).

The first reference genome sequence of *B. napus*, derived from the European winter‐type cultivar 'Darmor*‐bzh*', was previously published (Chalhoub *et al*., 2014) but largely incomplete due to the limitation of the read length available at the time. The ‘Darmor‐*bzh*’ genome sequence was assembled with NGS short reads and often contains numerous sequencing gaps with missing sequences or errors, which makes it difficult to be utilized for downstream applications. Subsequently, another European winter‐type cultivar 'Tapidor' was also assembled with NGS short reads, leading to a suboptimal quality assembly (Bayer *et al*., 2017). Additionally, a Chinese semi‐winter‐type cultivar 'Ningyou7' (NY7) with ‘double‐high’ traits (high erucic acid and high glucosinolate quality) was assembled from mostly NGS short reads, while the sequencing gaps were filled in by PacBio reads (Zou *et al*., 2019). An alternative ‘double‐low’ semi‐winter‐type *B. napus* cultivar 'ZS11' attracted the attention of the *B. napus* community with its high oil content and high seed production, with the first genome release (ZS11_NGS) completed based on a hybrid strategy using BAC clones and NGS short reads (Sun *et al*., 2017), resulting in a similarly lower quality assembly. Here, we reported a much‐improved assembly of genome (ZS11_PB) through a *de novo* assembly by integrating long PacBio SMRT reads, genetic maps and Hi‐C technologies. Our new *B. napus* genome assembly is shown to be more complete than both Darmor*‐bzh* and ZS11_NGS, as shown in a variety of completeness and contiguity metrics. Finally, we have annotated a near‐complete set of NLR genes owing to the high contiguity and completeness of our assembly, which offered a valuable resource for future genetic and disease resistance in *B. napus* and comparative genomic studies in *Brassicaceae*.

## Results

### Genome assembly

**Figure 1:**
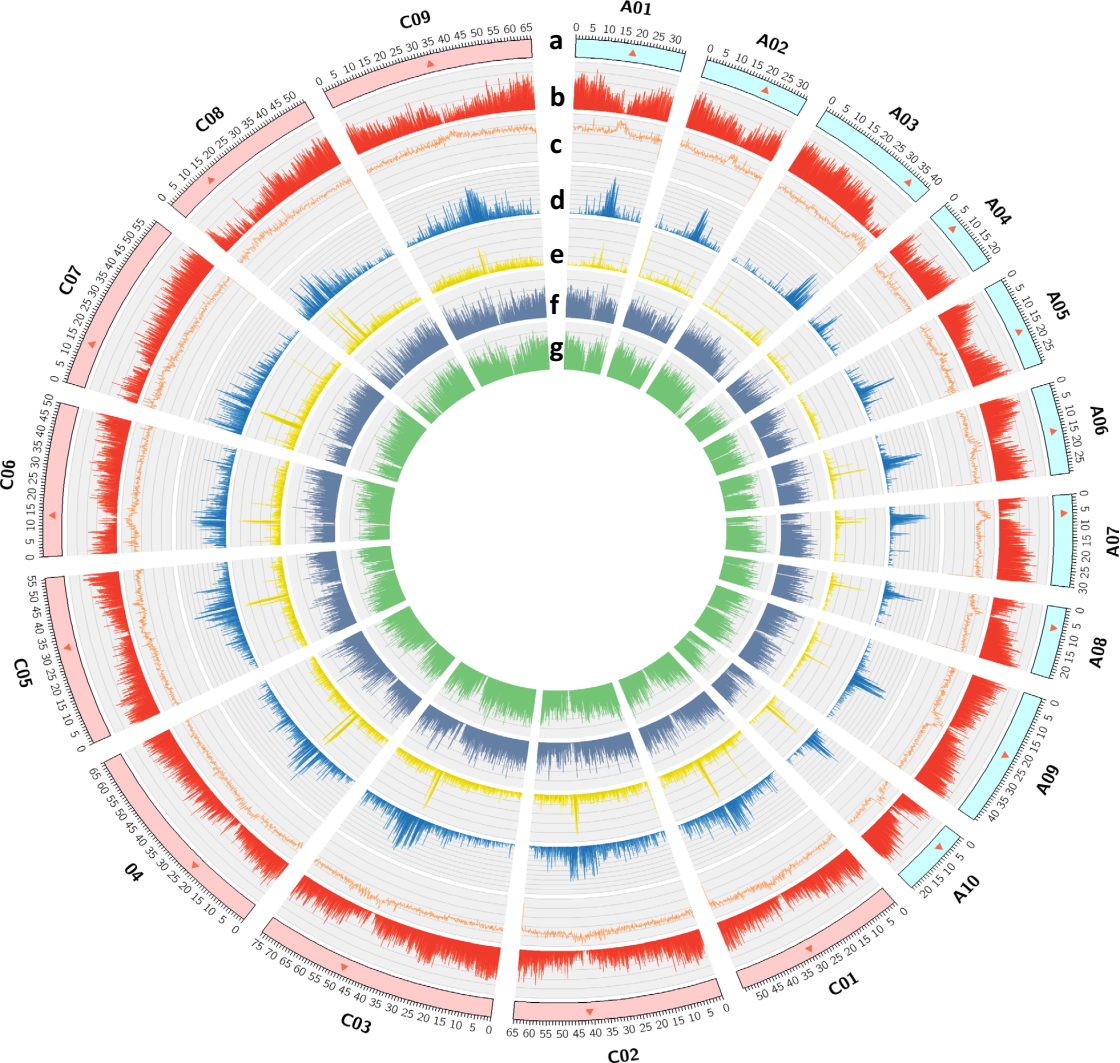
Genome features of the *B. napus* ZS11_PB genome assembly. (a) Distribution along the assembled 19 chromosomes (A01 to A10, and C01 to C09), red triangle shows the inferred position of centromeres based on the centromeric repeats. (b) Gene density track. (c) GC content track (non‐overlapping windows of 100 Kb). (d and e) The density of *gypsy* and *copia* retrotransposon. (f and g) Level of gene expression in buds and roots.

*B. napus* cultivar 'ZS11' was sequenced and assembled based on PacBio data (128 Gb, ~100× coverage), and the draft genome size of the initial assembly was 1.16 Gb, of which 921.5 Mb were a set of high‐confidence genomic sequences (highly continuous, high‐coverage, structurally accurate). Another ~240 Mb was predicted to be of lower confidence, which is enriched for hypothetical, partial and/or transposon‐related sequences, and possess shorter sequences than the high‐confidence set. The high‐confidence genomic sequence, 921.1 Mb, comprises 2404 contigs with a N50 size of 1.64 Mb, and maximum contig length of 12.1 Mb (Table S3). The initial assembly result was about 22‐, 32‐, 494‐ and 25‐fold more contiguous than the other released versions of four genomes (ZS11_NGS, Darmor*‐bzh*, Tapidor and NY7), whose N50 size of contigs was 55.5, 37.9, 3.4 and 69.0 Kb, respectively (Table 1, Table S3). Subsequently, Hi‐C data (100× coverage) were used to construct super‐scaffolds, and the four published genetic maps (DY, Z7, GP and TN) and ALLHiC pipeline (Zhang *et al*., 2019b) were used to organize 1403 scaffolds into 19 pseudochromosomes (Figure 1, Figures S1‐S2). The final assembly yielded a 921.5 Mb genome with a contig and scaffold N50 size of 1.64 and 53.2 Mb (Table S3). For this improved version, A and C subgenomes contained 310.6 Mb (33.7%) and 547.5 Mb (59.4%), respectively (Table S4). A total of 93.1% of the assembled sequences were anchored onto the 19 pseudochromosomes, with an anchor rate much higher than ZS11_NGS (87.6%, Table S4).

**Table 1:** Statistics of assemblies and annotation in *B. napus* genome

	ZS11_PB	ZS11_NGS	Darmor‐*bzh*
Assembly			
Longest (bp)	12 053 436	504 945	349 037
Number	4035	34 434	44 643
Total length (bp)	921 131 225	910 899 039	738 349 300
Mean contig length (bp)	228 285	26 453	16 538
Contig N50 (bp)	1 644 119	55 458	37 901
N50 number	160	4870	5477
GC content	36.90%	36.50%	36.10%
Annotation			
Total gene number	106 059	101 942	101 040
Average gene length (bp)	2731.07	2086.72	1953.13
Average CDS length (bp)	227.62	218.85	204.06
Average exon number per gene	5.34	5.57	5.47
Average exon length (bp)	278.48	229.48	212.62
Average intron length (bp)	286.89	229.49	176.87

### Validation of the assembly

We validated our *B. napus* 'ZS11' genome assembly (ZS11_PB) using a number of orthogonal metrics including read mapping rate, gene‐level and transcript‐level completeness, and overall concordance with Hi‐C mapping data. On read mapping rate, we mapped 50× Illumina short reads back to the assembled genome using BWA (v0.7.15) (Li and Durbin, 2009). A total of 99.65% of reads were mapped, and 98.49% were properly paired (Table S5).

On gene‐level completeness, BUSCO (v3.0) (Simao *et al*., 2015) program was carried out with default settings based on 1440 conserved plant genes (embryophyta_odb9). Overall, 98.3% of completeness were detected in the assembled genome that is slightly higher than ZS11_NGS (98%), Darmor‐*bzh* (97.6%) and Tapidor (94.8%), and same as NY7 (98.3%) (Table S6). Furthermore, CEGMA (v2.5) (Parra *et al*., 2007) showed 100% of completeness over the 248 Core Eukaryotic Genes (CEGs), which is also better than the other three genomes (ZS11_NGS, Darmor‐*bzh* and Tapidor) (Table S7). On transcript‐level completeness, 277 657 of 941 323 full‐length non‐chimeric reads remained after eliminating the redundancy sequences via CD‐HIT (v4.6) (Li and Godzik, 2006). Among them, 275 970 (99.39%) could be re‐mapped to the improved genome sequences by GMAP (version 2013‐10‐28) (Wu and Watanabe, 2005) with default settings (Table S8).

Finally, the genome‐wide interaction contact map based on HiC‐Pro (v2.9.0) (Servant *et al*., 2015) and HiCPlotter (v0.7.3) (Akdemir and Chin, 2015) showed that the intrachromosomal signal was notably stronger than interchromosomal signal, supporting a decent clustering within pseudochromosomes (Figure S3). We also observed that the Hi‐C contact matrix in ZS11_PB was much better supported than ZS11_NGS upon closer inspection, that is stronger diagonal signal and overall better signal‐to‐noise ratio (Figure S3). All of the above metrics indicated that the overall integrity and accuracy of our genome assembly are much higher than previous versions and are also more concordant with the genome mapping data (Figure S3).

### Repeat annotation and centromere identification

**Figure 2:**
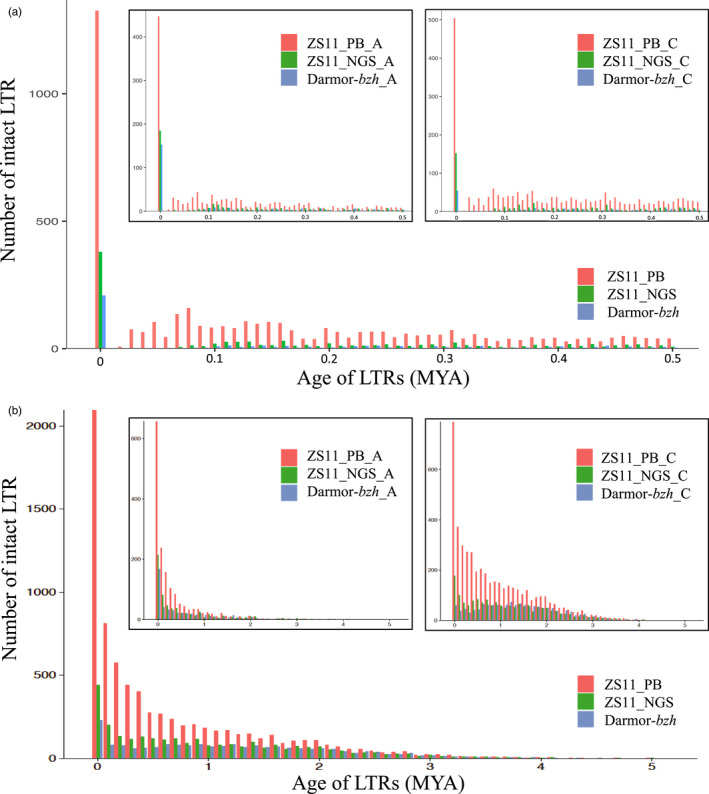
The number of intact LTR‐RTs with different insertion age in *B. napus* genomes. (a) The number of intact LTR‐RTs from 0 to 500 000 years ago. (b) 0–5 million years ago (MYA).

For a fair comparison, we annotated the repeat content in both of the ZS11_NGS and ZS11_PB assembled genome sequences with the same computational methods run in parallel. Transposable elements (TEs) explained 46.07% (449.79 Mb) and 61.83% (569.75 Mb) of ZS11_NGS and ZS11_PB genome sequences, respectively, and half of these sequences belong to retrotransposon families (258.65 Mb in ZS11_NGS, 285.19 Mb in ZS11_PB) (Table S9, Figure S4). In particular, the long terminal repeat (LTR) sequences account for 23.06% (212.49 Mb) of ZS11_PB genome, a higher percentage compared with the genomes based on NGS (17.84%, 174.20 Mb) (Table S9, Figure S5). We further detected the intact LTR retrotransposons (LTR‐RTs) *ab initio* and found that the ZS11_PB genome contains the largest number of LTR‐RTs, not only on the genome level but also on the subgenome level (Figure 2). In total, ZS11_PB has an additional 120 Mb (13.02% of ZS11_PB genome) sequences compared with ZS11_NGS, since SMRT sequencing reads can span many repetitive regions resulting in fewer sequencing gaps (Table S9).

In order to identify the centromere repeats, we used a similar approach, applied in *Oropetium thomaeum* (VanBuren *et al*., 2015) genome before, and found that the base centromeric repeat unit was 176 bp (Figure S6). The largest repeat arrays on each chromosome were identified, which were to be clustered into 19 centromeres (Table S10) with average length of 1.85 Mb. The centromeric and pericentromeric regions often contain clusters of retrotransposons *Gypsy* and *Copia* and were associated with lower gene density and higher GC content (Figure 1). Most LTR‐RTs were recent insertions with ‘burst of insertions’ dated back to ~2 MYA ago (Figure 2).

### The characteristics of recently amplified LTR‐RTs in *B. napus*

Repeat sequences in the A and C subgenomes increased significantly from 500 000 years ago, and also over the recent 100 000 years. The ZS11_NGS genome only saw an increase in the latter, but did not see an increase from 500 000 years ago. We examined the chromosomal distribution of LTR‐RTs and constructed the phylogenetic relationships among them. According to the ages of LTR‐RTs, we divided all LTR‐RTs into three different groups <0.2 My (Million years), 0.2–1 and >1 My. The young LTR‐RTs with age <0.2 My were dispersed across the length of the chromosomes but showed major peaks in centromeric and pericentromeric regions (Figure S7a). The LTR‐RTs from three different age groups are mixed in majority of clades, suggesting that these LTR‐RTs shared a common origin and continuously amplified through an extended period of time. However, we found that some specific clades (blue clades) only included LTR‐RTs with age <0.2 My (Figure S7b,c), suggesting a much more recent origin. Additionally, we found that 45% of LTR‐RTs with age <0.2 My were distributed in centromeric region, but this ratio was only 29% for LTR‐RTs with age 0.2–1 My and only 26% for LTR‐RTs with age >1 My (Table S11). These young and highly similar LTR‐RTs were significantly preferentially inserted into centromeric and pericentromeric region, which are challenging to assemble from short reads alone. As a result, these LTR‐RTs are poorly represented in ZS11_NGS. To compare the difference in LTR‐RTs between *B. napus* and the diploids *B. oleracea* and *B. rapa*, we further identified LTR‐RTs in *B. oleracea* and *B. rapa* genomes using the same methods applied in *B. napus*. *B. napus* was thought to have been formed in 5000–10 000 years ago through the hybridization of two diploid parental genomes (Chalhoub *et al*., 2014). It was found that 1327 of 7538 LTR‐RTs have identical pairwise sequences at long terminal ends of LTR‐RTs; therefore, their insertion time was estimated to be less than 10 000 years. These LTR‐RTs were probably originated from within the *B. napus* genome after polyploidization. For 1327 LTR‐RTs with age <10 000 years, we checked their presence and absence in the syntenic regions of diploid genomes by comparing LTR‐RTs themselves and their flanking regions of *B. napus* with those of *B. oleracea* and *B. rapa*. The results showed that 843 of 1327 LTR‐RTs in *B. napus* genome were confirmed to be species‐specific because they were absent in syntenic genomic location of *B. rapa* and *B. oleracea*. We checked the chromosomal distribution of these LTR‐RTs and found that they were widely dispersed along chromosomes but have two peaks in C04 and C08 (Figure S8a). For these two genomic regions, the LTR‐RTs exhibited preferential insertions in the syntenic region of *B. napus* and not found in the *B. oleraceae* counterpart (Figure S8b).

### Genomic annotation and TE‐related gene identification

A total of 106 059 gene models were obtained in ZS11_PB genome, approximately 4000–5000 more genes than the other two genomes. Compared with the other two published *B. napus* genomes, our gene models have a longer average gene length (2731 bp), CDS length (223 bp), exon length (278 bp) and intron length (287 bp) (Table 1). Then, we identified 15 384 (14.51%), 10 892 (10.68%) and 7271 (7.20%) TE‐related genes in ZS11_PB, ZS11_NGS and Darmor‐*bzh* genomes, respectively (Table S13). A total of 65 059 and 50 590 one versus one gene pairs were found between ZS11_PB vs ZS11_NGS and ZS11_PB vs Darmor‐*bzh*. Among them, 45 286 (69.60%) and 38 378 (75.86%) genes in ZS11_PB were longer than those of ZS11_NGS and Darmor‐*bzh* (Table S13). Among these gene pairs with long genes in ZS11_PB, TE‐related genes accounted for 5.78% (2616) and 4.91% (1886) in ZS11_PB, whereas this ratio is only 4.10% and 0.78% for ZS11_NGS and Darmor‐*bzh*, respectively (Table S13). Compared with ZS11_NGS, it was found that more TE‐related genes near the centromeres were identified in the A subgenome of ZS11_PB. In the C subgenome, the total length of chromosomes was longer and the number of TE‐related genes was significantly increased, particularly in chromosomes C05, C06, C08 and C09 (Figure S9). Besides, the TE‐related genes of ZS11_PB were mapped to ZS11_NGS and Darmor‐*bzh* genomes using BLAST (Camacho *et al*., 2009), and a total of 15 354 and 15 370 genes were anchored to the two genomes with 2244 (14.59%) and 4541 (29.52%) genes were distributed on scaffolds. Above results suggested that we had identified more TE genes and most of them were longer than their counterparts in ZS11_NGS and Darmor‐*bzh*, further illustrating the higher contiguity of our assembly and better resolution for TE‐related sequences.

Among the 106 059 gene models, 105 441 (99.42%) genes were distributed in A subgenome (44 373, 41.84%) or C subgenome (61 069, 57.58%) and only 617 (0.58%) gene scattered on unanchored scaffolds, compared with 8484 (8.32%) unanchored genes in ZS11_NGS (Table S4). We found that 76.48% (81 123) of the predicted genes had functional domains or GO (gene ontology) information and 97.34% (103 253) of genes could be annotated with potential functions (Table S14).

### Subgenome evolution

**Figure 3:**
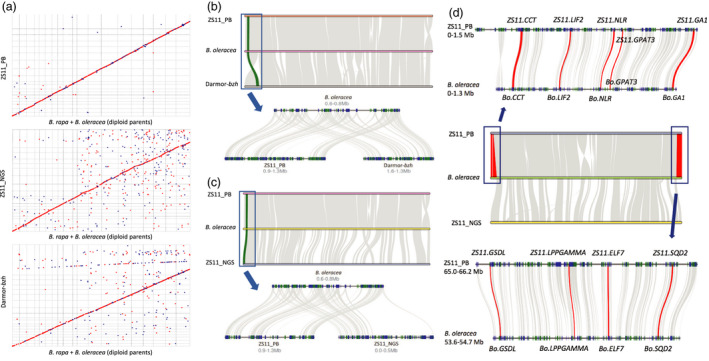
Comparative analysis between different genomes. (a) MUMmerplot comparison of the allopolyploid (*y*‐axis) and their diploid parents (*x*‐axis). Chromosome order on the *x‐* and *y*‐axis was both ordered from A01 to A10, and C01 to C09. Blue points indicate likely chromosomal inversions and breakage. (b and c) Macrosynteny (upper) and microsynteny (bottom) plots between allopolyploid and *B. oleracea* on the chromosome C02. The bottom half was a randomly selected region for illustration purpose. Grey wedges connecting the chromosomes represent the synteny blocks. Blue and green boxes represent for different genes, with plus or minus orientations, respectively. (d) Macrosynteny (middle) and microsynteny (upper and bottom) between ZS11 and their diploid parents *B. oleracea* on the chromosome C09. The red connecting wedges represent the synteny blocks between ZS11_PB and *B. oleracea,* which was misassembled in ZS11_NGS. The red lines represent a selected set of functionally important genes (Table S15).

We constructed the syntenic relationship between the tetraploid *B. napus* genomes and its diploid progenitor genomes. At the genome level, ZS11_PB has less noise and breakage than other two genomes (ZS11_NGS and Darmor‐*bzh*), which were both based on short‐read NGS sequencing (Figure 3a). More specifically, both ZS11_NGS and *Darmor‐bzh* have a larger number of unanchored sequences that show similarity to the progenitor genomes, while our assembly ZS11_PB has accurately placed them within their expected collinear positions (Figure 3a).

When zoomed into the scale of individual chromosomes, the synteny blocks between ZS11_PB and diploid parents also appear larger and more continuous, especially in the C subgenome (Figure S10). Local synteny views, as randomly inspected, also supported that the ZS11_PB presented much higher level of synteny against Darmor‐*bzh* compared with ZS11_NGS (Figure 3b‐3c). In many cases, we found that ZS11_NGS assembly showed chunks of likely misassembled sequences with functionally important genes such as flowering related genes (*CCT* gene, *ELF7* gene, *GA1* gene, *LIF2* gene), acyl‐lipid related genes (*GTAP3* gene, *GDSL* gene, *LPPGAMMA* gene, *SQD2* gene) and resistance genes (*NLR* gene), again highlighting the significance of a high‐quality reference assembly (Figure 3d, Table S15).

Neutral mutation rate (*Ks*) was calculated between the syntenic gene pairs to measure the time of divergence between the different *B. napus* subgenomes and their diploid parents. Compared with the diploid parents, *Ks* peak in the C subgenome were smaller than A subgenome indicating that the evolution rate of the C subgenome was slower than A subgenome (Student’s *t*‐test, *P* < 2.2e‐16) (Figure S11a). Species phylogeny was constructed using salt cress (*Eutrema salsugineum*) as outgroup, and the subgenome of different *B. napus* cultivars was clustered together (Figure S11b). Moreover, the gene function between the two subgenomes evolved in an asymmetric manner after polyploidization. In GO (gene ontology) analysis, there were 122 and 488 significantly enriched GO terms in the A and C subgenomes (*P *≤ 0.05), respectively. After statistical tests and FDR correction, 42 and 189 significantly enriched GO terms were obtained in the A and C subgenomes, respectively (Figure S12, Table S16). Similarly, 11 and 8 specific KEGG (Kyoto Encyclopedia of Genes and Genomes) pathways were significantly enriched in A and C subgenomes (*P *≤ 0.05), and only 5 KEGG pathways were significantly enriched in C subgenome after FDR correction (Figure S12, Table S16). GO and KEGG enrichment analysis of ZS11_PB suggested that the A subgenome contains more genes associated with plant defence and oil accumulation (regulation of phospholipase A2 activity, fatty acid elongation, plant–pathogen interaction, fatty acid metabolism), whereas developmental related genes were dominant in the C subgenome (integument development, plant ovule development, flower development, floral organ development) (Figure S12, Table S16).

### Resistance (R)‐gene annotation and the identification of NLR gene pairs

Due to the preference of tandem duplication of the nucleotide‐binding leucine‐rich repeat (NLR) genes (van Wersch and Li, 2019), which are typically located in regions with many similar NLR genes, these genes are often intractable for the NGS method while PacBio SMRT sequencing may overcome these issues. The NLR genes are also notoriously more difficult to annotate (Bayer *et al*., 2018), prompting us to use a more exhaustive and accurate method to re‐annotate the NLR genes.

**Figure 4:**
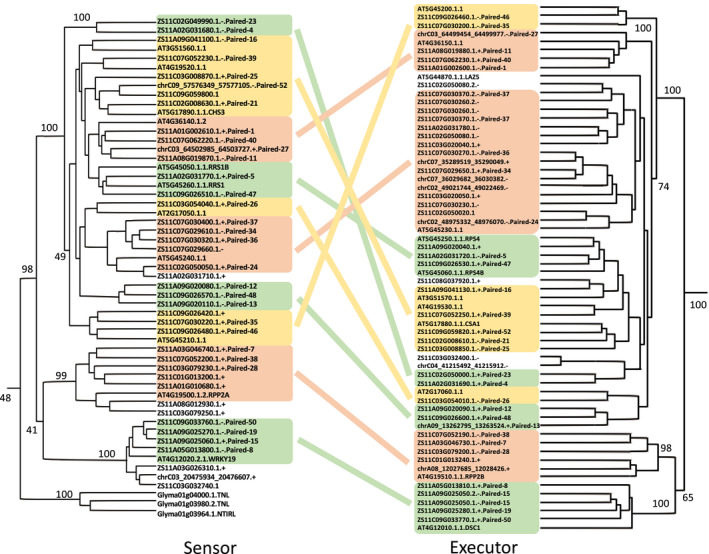
Maximum‐likelihood phylogenetic trees of ‘sensor–executor’ paired NLR genes identified in ZS11_PB genome. Paired NLR genes (two NLR genes shared an upstream region in opposite directions (van Wersch and Li, 2019)) in the same subclade are linked by the same colour. The paired NLR genes in ZS11_PB were marked with 'Paired', and the sensor–executor paired NLR genes in *A. thaliana* were identified in (Van de Weyer et al., 2019). The symbols '+' and '‐' represent the plus or minus orientations of the NLR genes.

We first identified 419 NLR genes (166 and 253 genes in A and C subgenome, respectively) in *B. napus* based on genome annotation using PfamScan (v1.6) (Finn *et al*., 2014) with default settings (Table S17). In order to annotate more divergent NLR genes, a similar method described in sugar beet (Funk *et al*., 2018) was adopted in our study. We built the nucleic acid seeds representing nucleotide‐binding ARC (NB‐ARC) domains and subsequently used these seeds as a more sensitive method to search against the assembled genome to infer candidate disease resistance loci. A total of 569 NB‐ARC tentative loci were identified in ZS11_PB (217 and 351 genes in A and C subgenome, respectively). Finally, we combined these gene models with the starting set of NLR gene, and finally, 597 NLR genes were identified. Of the 597 NLR genes, 425 were identified based on the original set, 144 were absent in the original set, while 28 were not annotated by our method (Table S17a,b, Figure S13), suggesting that our method has better sensitivity for the discovery of NLR genes. As an informal validation, these tentative loci are clustered with known NLR genes, further suggesting that they are indeed coding NB‐ARC domain (Figure S14). In addition, the head‐to‐head NLR gene pairs were identified according to the method described in '*Tetep*' rice genome (Wang *et al*., 2019a). The unique arrangement forms a ‘sensor–helper’ pair (Wang *et al*., 2019a), known in *Arabidopsis thaliana* as a ‘sensor–executor’ pair (Van de Weyer et al., 2019). The sensor functions as decoy with an integrated domain (ID), which interacted with the signalling executor (helper) to trigger defence response (Liang *et al*., 2019; Ma *et al*., 2018; Narusaka *et al*., 2009; Saucet *et al*., 2015; van Wersch and Li, 2019; Xu *et al*., 2015). We found 52 NLR gene pairs (17.4% of NLR genes) in the ZS11_PB genome, of which 30 pairs were mainly clustered in two clades that corresponded to the NLR gene pairs in *A. thaliana* (Van de Weyer *et al*., 2019) (Figure 4, Table S18, Figure S14). These findings confirmed that our method is effective, both in terms of sensitivity and specificity, supporting our claim that the prediction of the NLR genes cannot rely solely on wholesale genome annotation without additional curations.

### Selective sweeps between rapeseed ecotypes

Based on the new assembled genomic sequences, selective sweeps and phylogenetic analysis were performed using previously published resequencing data of 991 *B. napus* accessions (658 winter, 145 semi‐winter, 188 spring) (Wu *et al*., 2019a). First of all, we identified 18 759 245 single nucleotide polymorphisms (SNPs) (6 468 179 SNPs were located in TE regions) with 50 032 864 effects (one SNP can have multiple annotations). Among them, exons, introns, intergenic regions, upstream region and downstream region accounted for 8.60% (4 302 918), 6.65% (3 327 902), 22.33% (11 169 798), 29.26% (14 638 349) and 29.60% (14 808 978) of total effects, respectively. In addition, 1 731 530 and 2 529 420 SNPs within genes were annotated as either mis‐sense or synonymous variants (Table S19). Furthermore, a total of 51 477 (0.27%) SNPs were identified within the 597 NLR genes that we have curated in this study.

**Figure 5:**
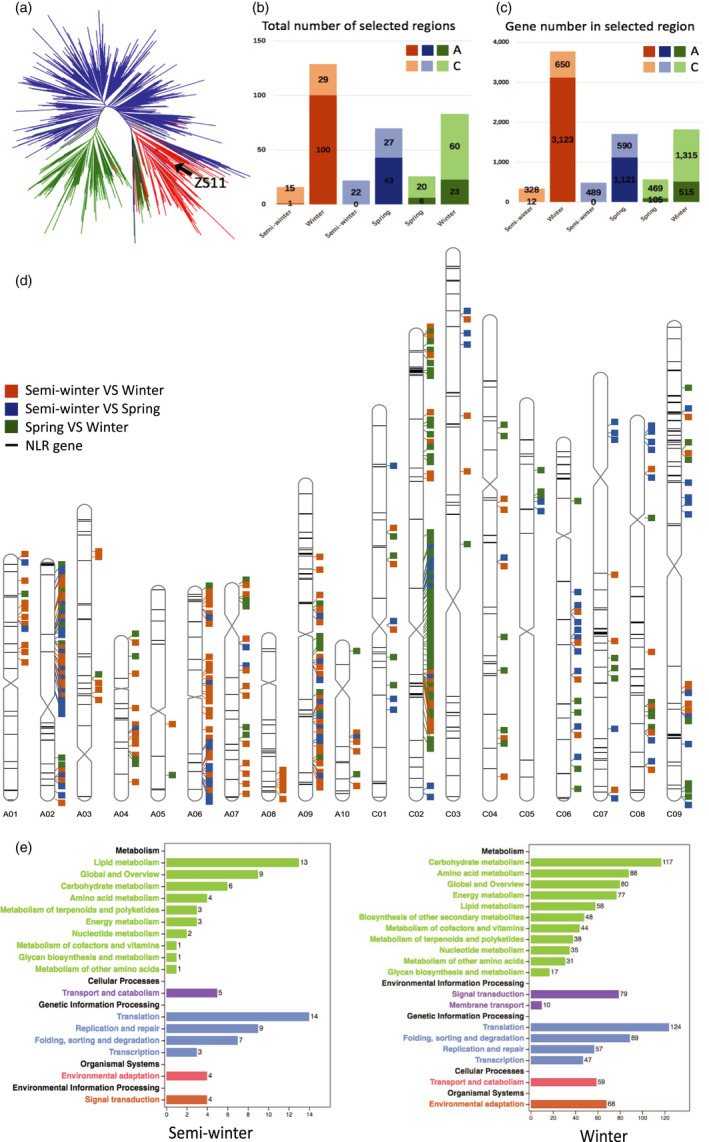
Phylogenetic relationships and the selective signal between different *B. napus* ecotypes. (a) A maximum‐likelihood (ML) tree. Blue, green and red represent winter, spring and semi‐winter ecotypes, respectively. The black arrow indicates the phylogenetic position of ZS11_PB. (b‐c) The total number of selective sweep regions (b) and genes (c) in different ecotypes. Orange, blue and green mean the comparative within semi‐winter VS winter, semi‐winter VS spring and spring VS winter. The light and dark colours represent the distribution in A and C subgenomes, respectively. (d) Distribution of NLR genes and selective sweeps between different *B. napus* ecotypes. Orange, blue and green mean the comparative within semi‐winter VS winter, semi‐winter VS spring and spring VS winter. (e) KEGG pathway annotation in the selected regions between semi‐winter and winter ecotype groups. The black labels on the *y*‐axis represent KEGG B class, the coloured label represents the specific pathway under the class, and the *x*‐axis shows the number of genes in a given KEGG pathway (Table S21).

Subsequently, the SNPs in the CDS region (with missingness < 2% of the genotyped accessions) were used to construct a phylogenetic tree and infer population structure (Figure 5a, Figure S15), the results showed that different ecotype groups were clustered together according to the broad classification of ecotype groups and growth habit. Our cultivar ZS11 is located in the cluster of semi‐winter, as expected (Figure 5a).

Selective sweeps were identified within the three ecotype groups based on the identified SNP sites. Firstly, we set the same threshold as the published data (highest 1% of the F_ST_) (Wu *et al*., 2019a) and found more selected regions and genes based on ZS11_PB genome (Table S20a). Here, we defined ‘selective sweeps’ as the set of genomic regions that contain sites with highest 5% value of F_ST_ and the lowest or highest 5% of π ratio between ecotype groups (see Methods). Naturally, these regions represented the regions that are highly differentiated in terms of allele frequencies between groups – suggesting likely selections in either genome (Li *et al*., 2013).

### Selective sweeps analysis between semi‐winter and winter ecotypes

Between semi‐winter and winter ecotypes, a total of 16 and 169 selective regions were detected covering 3.49 Mb (328 genes) and 31.98 Mb (3800 genes), respectively (Table S20b). Only one of the 16 regions located in A subgenome (0.1 Mb, 12 genes) for semi‐winter ecotype, while in the winter ecotypes, 100 of 169 regions were identified in the A subgenome with a cumulative length of 21.2 Mb (3123 genes) (Figure 5b‐5d and Table S20b). Additionally, among 3800 genes in winter ecotypes, three genes (*ZS11A02G022260, ZS11A02G022280* and *ZS11A09G044850*) corresponding to *FAB1* gene (*BnaA02g17050D*)*, LPAT4* gene (*BnaA02g17090D*) (Qu *et al*., 2017) and *MYB44* gene (*BnaA09g35310D*) (Wu *et al*., 2019a) in Darmor‐*bzh* were found to be associated with fatty acid biosynthesis and flowering time based on previous GWAS analysis (Table S23). From the KEGG analysis, we found that lipid metabolism pathway and environmental adaptation pathway were both enriched among the 3800 genes in the semi‐winter ecotypes (Figure 5e, Table S21).

### Selective sweeps analysis between semi‐winter and spring ecotypes

There were 29 and 75 regions that spanned 5.43 Mb (489 genes) and 13.61 Mb (1714 genes) for semi‐winter and spring ecotypes, respectively (Table S20b). In semi‐winter ecotypes, all the selective sweeps were located in the C subgenome (22 regions, 4.73 Mb, 489 genes) and super‐scaffolds (7 regions, 0.7 Mb, no genes). For spring ecotypes, 43 and 27 regions were identified in the A (7.7 Mb, 1121 genes) and C (5.32 Mb, 590 genes) subgenome, respectively (Figure 5b‐5d, Table S20b). Spring ecotypes had a larger percentage of genes in the environmental adaptation pathway according to KEGG terms (Figure S16a, Table S21).

### Selective sweep analysis between spring and winter ecotypes

We identified 27 and 135 regions between spring and winter ecotypes, harbouring a total of 5.01 Mb (574 genes) and 25.63 Mb (1839 genes), respectively. The A and C subgenome contained 6 (0.81 Mb, 105 genes) and 20 (4.1 Mb, 469 genes) regions in spring ecotypes, while 23 (3.42 Mb, 515 genes) and 60 (16.36 Mb, 1315 genes) regions in winter ecotypes (Figure 5b‐d and Table S20b). In the selective sweep regions of the winter ecotypes, *ZS11A05G036270* and *ZS11A02G036710* were the orthologues of *MYB83* gene (*BnaA05g29680D*) and C*RF11* gene (*BnaA02g27680D*) that were previously shown to be associated with yield and flowering time, respectively (Lu *et al*., 2017; Wu *et al*., 2019a) (Table S25). KEGG result showed that spring ecotypes had more lipid metabolism and fewer environmental adaptation pathway genes than winter ecotypes (Figure S16b, Table S21).

### The rapid evolution of NLR genes in different *B. napus* ecotypes

We extracted the nucleotide sequences from the published QTL regions related to three major diseases (Fu *et al*., 2019) and mapped the sequences back to ZS11_PB genome to identify the corresponding QTL regions using MUMmer (v3.5) (Delcher *et al*., 2003). We were able to unambiguously identify 70 out of the 71 QTL regions in ZS11_PB (Table S22). There are many new annotated NLR genes in selected regions and QTL regions; for example, there are three new annotated NLR genes that were identified in the blackleg resistance region chrC07 36.83–41.24 Mb (Table S22). One region contains a previously annotated NLR gene, but we have annotated additional copies of the NLR genes in this region, such as one of the selected regions winter‐25 (one NLR gene, 4 new annotated NLR genes) (Table S25).

**Figure 6:**
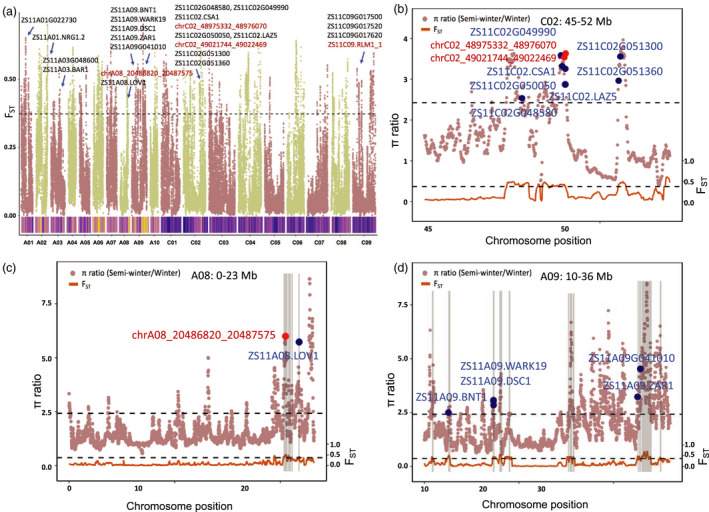
Disease resistance genes within the selected regions between semi‐winter and winter ecotypes in *B. napus*. (a) The F_ST_ distribution and density of π ratio (semi‐winter/winter) between semi‐winter and winter ecotype groups. The upper part represents the distribution of F_ST_ in each chromosome, and the top 5% of F_ST_ values drawn in black dotted line. The lower part represents the distribution of the π ratio, and blue and yellow mean the selective sweep region in semi‐winter and winter ecotypes, respectively. All the NLR genes were labelled. The combination of extremal values of FST and π together defines ‘selective sweeps’ in this study. (b‐d) F_ST_ and π ratio on the chromosome C02 (b), A08 (c) and A09 (d). The black dotted line represents the top 5% of π ratio and F_ST_ values. The grey interval represents the selected region that overlaps with the identified QTL regions. The blue point indicates the locations of the predicted NLR genes, and red point indicates the locations of the new annotated NLR gene. (b) C02, none of the NLR genes overlapped with the identified resistance QTL regions. (c‐d) A08, A09, all the NLR genes were located on the identified resistance QTL regions.

We further identified many NLR genes specific to different *B. napus* ecotypes by investigating their distribution in selective sweep regions in different ecotypes. We identified 24 NLR genes (including 4 new annotated NLR genes) for winter ecotypes, while no NLR genes were found for semi‐winter ecotypes in the selected regions (Figure 6, Table 2). Among these, 10 NLR genes were homologous to the *A. thaliana* functional NLR genes, such as *NRG1.2* gene (Wu *et al*., 2019b), *BAR1* gene (Laflamme *et al*., 2020) and *BNT1* gene (Sarazin *et al*., 2015; Warmerdam *et al*., 2020) (Table S26). Moreover, three NLR gene pairs (*ZS11A09.DSC1/ZS11A09.WARK19*, *ZS11C02G049990/ZS11C02.CSA1* and *chrC02_48975332_48976070/ZS11C02G050050*) were identified in the winter selected region and *ZS11A09.WARK19, ZS11C02G049990* and *ZS11C02G050050* were clustered in the sensor clade, while *ZS11A09.DSC1, ZS11C02.CSA1* and *chrC02_48975332_48976070* (named by chromosome with domain position) in the executor clade (Figure 4, Tables S18 and S26). The homologous gene pair of ZS11_PB (*ZS11A09.DSC1/ZS11A09.WARK19*) in *A. thaliana* (*AT4G12010.DSC1/AT4G12020.WRKY19*) has been shown to be associated with the root‐knot nematode *Meloidogyne incognita* (Warmerdam *et al*., 2020).

**Table 2:** Summary of selective sweeps between the three ecotypes of *B. napus*

Ecotype groups	Selected ecotype	Gene number ^1^	NLR gene number (new annotated) ^2^	Known NLR genes (new annotated) ^3^
Semi‐winter vs Winter	Semi‐winter	340	0	0
	Winter	3800	24 (4)	11 (1)
Semi‐winter vs Spring	Semi‐winter	489	0	0
	Spring	1715	12 (4)	2 (1)
Spring vs winter	Spring	574	0	0
	Winter	1839	13 (8)	12 (7)
Total	7165	49 (16)	25 (9)	

1. The total number of genes in selected ecotype. 2. The number of NLR genes in selected ecotype. 3. The number of known diseases of NLR genes in selected ecotype.

In addition, 2 of 12 predicted NLR genes (including 4 new annotated NLR genes) were identified to specially exist in spring ecotypes, and none in the semi‐winter ecotypes (Table S24). 5 of the 12 NLR genes were homologous with *A. thaliana* functional NLR genes (*DAR5* gene, *RLM1* genes (Persson *et al*., 2009), *RSP6* gene (Gloggnitzer *et al*., 2014) and *VICTL* gene (Kim *et al*., 2012)), and one NLR gene pair (*ZS11C09.VICTL/ZS11C09G027620*) was found in the spring‐66 selected region (Tables S18, S24, S26).

Similarly, none of NLR genes in the selected regions of spring ecotypes, but 13 predicted NLR genes (including 8 new annotated NLR genes), were specific to the winter ecotypes, which are only distributed on the chromosome A09 and C02 (10 of the 11 NLR genes on chromosome C02 were clustered into the subclade with *AT5G18350* and *AT5G18370.DSC2* gene in *A. thaliana*), and almost all of them (12 of 13 NLR genes) overlapped with the reported QTL resistance regions (Table 2, Figure S14, Tables S25‐S26). We also identified one NLR gene pair (*ZS11A09G041100/ZS11A09G041130*) in the winter‐20 selected region, which may be related to blackleg and *Sclerotinia* stem rot (Figure 4, Tables S22, S25), and they were clustered in sensor and executor clade, respectively (Figure 4). The above identified diversification of NLR genes in different ecotypes suggested that R‐gene evolved rapidly during the domestication and genetic improvement of *B. napus*.

## Discussion

In this study, we reported a high‐quality reference genome assembly of allotetraploid *B. napus* cultivar 'ZS11' integrating multiple data types including PacBio, Hi‐C and genetic maps (Figure S1). We assembled a total genome size of 1.16 Gb (the estimated size was 1345 Mb for Darmor‐*bzh* and 1335 Mb for Tapidor based on k‐mer composition (Bayer *et al*., 2017)). Our new ‘ZS11’ assembly achieved a significant increase in contig and scaffold N50 size and an improved accuracy based on the Hi‐C contact map compared with all previous assemblies (Sun *et al*., 2017) (Table 1, Table S3, Figure S3). Repeat annotation showed that ZS11_PB had 120 Mb sequences more than ZS11_NGS, most of which were interspersed repeats including LTR retrotransposons (Figure 2, Table S9, Figures S4‐S5). An intact list of 19 identified centromeres was also inferred in this study, highlighting the ability of long‐read sequencing in resolving repetitive regions and complex polyploid genome (Table S10). Complete and high‐quality reference assemblies, such as our improved ZS11 assembly, serve as a solid foundation for studying selection and domestication and facilitating the discovery of functional genes involved in important agronomic traits such as disease resistance.

The completed and high‐quality ZS11_PB genome sequences offered a good opportunity to investigate the distribution and evolution of LTR‐RTs. Similar to other plant genomes (Bennetzen and Wang, 2014), the insertion time distribution of LTR‐RTs follows an exponential decay and exhibits an L‐shaped pattern for *B. napus*. LTR‐RTs have experienced sequential bursts of amplification during the past 4 My, and have accelerated in the most recent 500 000 years. In ZS11_PB, we identified a large number of young LTR‐RTs, which preferentially accumulated and clustered around centromeres and pericentromeres (Table S11). Through syntenic comparison, we confirmed that the majority of young LTR‐RTs were specific to only *B. napus* but absent in the counterpart regions in the diploid progenitor genomes of *B. rapa* and *B. oleracea*. Overall, we have found clear evidence supporting the amplification of TEs during the initial allopolyploidization, which led to the formation of the *B. napus* genome and the diploidization afterwards. All of these discoveries may shed further light on the structural and epigenetic modifications of the *B. napus* genome, as driven by TEs. Additionally, the TE difference may also explain some of the trait differences between different *B. napus* cultivars.

Compared with the previous genome, we have annotated 597 NLR genes, all of which were encoded by the NB‐ARC domain, further demonstrating the advantage of long‐read sequencing. A recent study identified 1749 resistance gene analogues (RGAs) based on pangenome, comprising 503 NBS‐encoding genes and TX (TIR domain with unknown domain), 148 RLPs (membrane‐associated receptor‐like proteins) and 1098 RLKs (surface‐localized receptor‐like protein kinases) (Dolatabadian *et al*., 2020 ), while all of our curated 597 NLR genes were encoded by the NB‐ARC domain, which exceeded the aforementioned study (Table S17). Our careful and exhaustive re‐annotation of disease resistance genes on the high‐quality reference genome of *B. napus* facilitates functional characterization of these genes, since NLR genes are notoriously difficult to assemble due to their tendency for tandem duplication, as well as highly challenging to annotate accurately (Bayer *et al*., 2018).

Winter ecotype was the original form of *B. napus*, then diversified into the spring and semi‐winter ecotypes, while semi‐winter ecotype was the youngest lineage among the three ecotypes (Lu *et al*., 2019). According to the resequencing data of 991 *B. napus* accessions (Wu *et al*., 2019a), we have detected a lot of selective sweep regions among the three ecotype groups, in which five Darmor‐*bzh* orthologous genes were identified, which have been reported to be associated with important agronomic traits such as yield, flowering time and fatty acid biosynthesis (Tables S22‐S26) (Lu *et al*., 2017; Qu *et al*., 2017; Wu *et al*., 2019a). In particular, compared with spring and winter ecotypes, whose selective sweeps located at A subgenome, the selective sweeps of semi‐winter ecotypes are almost in the C subgenome. However, between spring and winter ecotypes, the C subgenome covered most of the selective sweeps, supporting an asymmetric pattern of subgenome evolution (Table S20b).

Moreover, we curated the most comprehensive list of disease resistance genes in a *B. napus* genome thus far and carefully analysed these genes that are located in selected regions. We found that there were differences in the number of resistance genes between the three ecotype groups, with winter > spring > semi‐winter (Table 2). Compared to the reported resistance QTL regions (Fu *et al*., 2019), all of the selected NLR genes in winter or spring ecotypes were acting against blackleg, *Sclerotinia* stem rot or both (Tables S22‐S26). The result showed that NLR genes exhibited diversity in different *B. napus* ecotypes suggesting that they have experienced fast evolution during the domestication and crop improvement of *B. napus*. This is consistent with the adaptation of the three ecotypes to their respective geographical environments and dominant types of local biotic stress.

It has been reported in *A. thaliana* and rice that NLR gene pairs with head‐to‐head arrangement could play their roles together (Van de Weyer *et al*., 2019; Van Wang *et al*., 2019; Wang *et al.,* 2019a): one member of a pair as a sensor and the other known as an executor. In our study, we identified 52 pairs of NLR genes, of which five pairs were located in the selected regions (Tables S18, S22‐S25). Moreover, four of the five pairs were clustered in two clades, which corresponded to sensor and executor clade in *A. thaliana* (Van de Weyer *et al.,* 2019), respectively (Figure 4, Table S22–S26). Among these, the homologous gene pair of ZS11_PB (*ZS11A09.DSC1/ZS11A09.WARK19*) in *A. thaliana* (*AT4G12010.DSC1/AT4G12020.WRKY19*) was reported to contribute to the resistance towards the root‐knot nematode *M. incognita* (Warmerdam *et al*., 2020).

An integrated strategy combining PacBio long‐read sequencing and chromatin conformation capture (Hi‐C) technology has substantially improved the genome assembly, and, in turn, also improved gene annotations of *B. napus*. An ‘upgraded’ *B. napus* genome reference and more precise sequences for functional genes will serve to refine association genetics, QTL mapping and further facilitating genetic and resistance breeding in oilseed rape. By cross‐referencing the selection signals against genome‐wide association signals, we have well demonstrated that pathogen resistance genes contributed to the divergent selection among the three ecotypes and has left footprints during the past domestication, breeding and improvement of various resistant cultivars of *B. napus*.

## Methods

### Sample collection and sequencing

### Material collection

The seeds of *B. napus* cultivar 'ZS11', the same accession used for 'ZS11_NGS', were collected from Oil Crops Research Institute, Chinese Academy of Agricultural Sciences in the city of Wuhan, Hubei Province, China.

### Genome sequencing

#### Illumina short‐read sequencing

A total of 73.3 Gb of clean data were downloaded from the previously released genome version of *B. napus* 'ZS11' (Sun *et al*., 2017), representing ~ 61× coverage of the *B. napus* genome.

#### PacBio library construction and sequencing

About 20 Kb SMRTbell libraries were prepared according to the manufacturer's protocols provided by PacBio Company. A total of 13 SMRT cells were sequenced on PacBio Sequel platform, and 128 Gb (~100 × coverage) of long reads was generated.

#### Hi‐C library construction and sequencing

Hi‐C library was created from young leaves of ZS11, which were digested with DpnII restriction enzyme. A total of 121.8 Gb (~100 × coverage) of clean data were sequenced by Illumina HiSeq 4000 platform with 150‐bp reads length with a 200‐bp insert size. All sequence data are summarized in Table S1.

### RNA sequencing

RNA sequencing was performed using both short and long reads, a strategy that was shown to provide a combination of completeness and accuracy of the transcriptome. For short RNA‐seq reads, total RNA was isolated from the bud, callus, leaf, silique and root tissues using the QIA‐quick PCR Extraction Kit (Qiagen) and three biological replicates were sampled for each tissue. Paired‐end sequencing reads of 150 bp in length were generated on the Illumina HiSeq 2000 system, yielding 296 Gb of clean data (Table S2). For Iso‐Seq long reads, 31 SMRT cells were sequenced using PacBio Sequel system that produced 41.08 Gb of raw data. The raw data were further classified into 2.4 Gb of full‐length non‐chimeric reads through CCS based on the Iso‐Seq2 pipeline. Potential sequencing error was corrected by LoRDEC (Salmela and Rivals, 2014), and the redundancy sequences were removed using CD‐HIT (Li and Godzik, 2006). Finally, we obtained 704.3 Mb of mostly full‐length transcript sequences with average length of 2527 bp.

### Genome assembly

The full Sequel subreads bam files were transformed into fastq format using Bedtools (v2.25.0) (Quinlan and Hall, 2010) package. Then, all the extracted reads were assembled by Canu (v1.5) (Koren *et al*., 2017) software with default parameters. The initial high‐quality assembly was about 921.12 Mb, and we also assembled 240‐Mb low‐quality sequences from the unassembled FASTA output file. For more accurate analysis, the further analysis was based on the high‐quality assembly. The primary high‐quality assembly was further polished by Pilon (v1.22) (Walker *et al*., 2014) with the 61 × Illumina reads (Figure S1). Hi‐C reads and ZS11_PB (Initial) were subjected to the 3D *de novo* assembly (3D‐DNA) pipeline (Dudchenko *et al*., 2017). We used Hi‐C scaffolding in order to reduce the misjoins in the input scaffolds and output the 'megascaffold' (or 'super‐scaffolds' sometimes referred to in other studies). Four published genetic maps including 'GH06' × 'P174'(GP) (Liu *et al*., 2013), 'Darmor‐*bzh*' × 'Yudal' (DY) (Chalhoub *et al*., 2014), 'ZS11' × '73290' (Z7) (Li *et al*., 2014) and 'Tapidor' × 'Ningyou7' (TN) (Zhang *et al*., 2016) were collected and formatted as input to ALLMAPS (v0.7.3) (Tang *et al*., 2015) to construct chromosomes (Figure S2) with an equal weight given to each of the 4 maps. Finally, an optimized Hi‐C ordering and orientation algorithm of ALLHiC (Zhang *et al*., 2019b) package was performed to improve the ordering and orientation based on Hi‐C data (Figure S1).

### Repeat annotation

Transposable elements (TEs) and a *de novo* repeat library were identified before protein‐coding gene model prediction. At the first step, RepeatModeler (http// www.repeatmasker.org/RepeatModeler/) was used to build a *de novo* repeat library. Subsequently, the above consensus TE sequences were used to discover and cluster the known TEs via RepeatMasker (v4.05, http//repeatmasker.org/). For unknown TEs, TE class (v2.1.3) (Abrusan *et al*., 2009) was carried out to categorize them. Additionally, the Tandem Repeat Finder (TRF) package (v4.07) (Benson, 1999) was executed to find the tandem repeat in the assembled genome sequences with the following settings '1 1 2 80 5 200 2000 ‐d ‐h'. The intact long terminal repeat (LTR) retrotransposons were *de novo*‐detected by LTR_Finder (v1.05) (Xu and Wang, 2007) with the settings of '‐D 15000 ‐L 7000 ‐C ‐M 0.9' and LTRharvest (v1.5.10) with the following parameters '‐seed 20 ‐minlenltr 100 ‐maxlenltr 7000 ‐similar 90 ‐mintsd 4 ‐maxtsd 6 ‐motif TGCA ‐motifmis 1 ‐vic 10'. Additional filtering was applied using LTR_retriever (Ou and Jiang, 2018) considering a set of structural and sequence features including target site duplications, terminal motifs and LTR‐RT Pfam domains. The insertion time of LTR‐RTs was estimated by comparing the sequence divergence of pairwise LTR‐RT sequences at both ends of each LTR‐RT. The following formula T = K/2r was used to calculate the insertion time for each LTR‐RT, where T is the insertion time, K is the genetic distance, and r is the base substitution rate. A mutation rate of substitutions per base per year of *r* = 1.4 × 10^−8^ was adopted (Cai *et al*., 2018).

### Genome annotation

Gene prediction was conducted via two rounds of MAKER (v2.0) (Campbell *et al*., 2014) and the Program to Assemble Spliced Alignments (PASA) (Haas *et al*., 2008) pipeline. Firstly, different NGS‐based RNA sequencing assembled strategies were adopted to assess the transcript evidence including StringTie (v1.3.3b) (Pertea *et al*., 2016) and PASA, which could integrate the *de novo* assembly and genome‐guided assembly by Trinity (v2.2.0) (Haas *et al*., 2013). BUSCO (v3.0) (Simao *et al*., 2015) analysis was executed to evaluate the completeness containing the full‐length transcript sequences described previously. Finally, we choose the Iso‐Seq‐PASA as transcript evidence, which has the highest completeness (97.4%) as shown in Table S12. The *ab initio* predictions of AUGUSTUS (v3.2.2) (Stanke *et al*., 2006) software trained by full‐length transcript and GENEMARK (v4.32) (Lomsadze *et al*., 2005) models were obtained using BRAKER (v1.8) (Hoff *et al*., 2016). Protein repertoires of plants including *A. thaliana TAIR10, Carica papaya ASGPBv0.4, Populus trichocarpa v3.1, Vitis vinifera Genoscope.12X* (download from Phytozome 12 https//phytozome.jgi.doe.gov/pz/portal.html#), *B. juncea (GCA_001687265.1), B.napus* Darmor*‐bzh* (Chalhoub *et al*., 2014), *B. rapa* (Wang *et al*., 2011), *B. oleracea* (Parkin *et al*., 2014) and SWISS‐PROT (September 2017) were used as evidences of homologous proteins. All evidences were imported to MAKER (Campbell *et al*., 2014) pipeline for the first round of constructing an integrated gene model. Predicted genes with annotation edit distance (AED) scores greater than 0.25 were filtered for re‐training using SNAP (v2006‐07‐28) (Korf, 2004) and AUGUSTUS (v3.2.2) (Stanke *et al*., 2006). A second round of MAKER was run with this filtered gene model set for better performance. Finally, these gene models were submitted to PASA pipeline to correct exon boundaries, add UTRs and alternatively spliced models and generate an updated gene set. The corrected gene models were clustered by OrthoFinder (v2.2.6) (Emms and Kelly, 2015) with *B.rapa* (Wang *et al*., 2011), *B.oleracea* (Parkin *et al*., 2014), ZS11_NGS (Sun *et al*., 2017) and Darmor‐*bzh* (Chalhoub *et al*., 2014) genome. Finally, we removed the unassigned genes in OrthoFinder or encoding less than 50 amino acids and lacking transcripts support to generate a high‐confidence annotated gene set. The BUSCO test was used to evaluate the quality of our annotation with 98.9% of complete gene models that was slightly higher than the other two genomes (Table S6).

After that, five different databases were applied to functional annotation of ZS11_PB, including the PfamScan (v1.6) (Finn *et al*., 2014), InterProScan (v5.30) (Jones *et al*., 2014), KEGG Automatic Annotation Server (KAAS, https// www.genome.jp/kegg/kaas/), EggNOG (v4.5.1) (Huerta‐Cepas *et al*., 2016) and homology against the NCBI NR database (https// www.ncbi.nlm.nih.gov/) using BLASTP (v2.2.28) (Camacho *et al*., 2009) package with default settings.

For a side‐by‐side comparison between ZS11_PB and ZS11_NGS, reciprocal blast was carried out using BLASTP (v2.2.28) (Camacho *et al*., 2009) with the parameters of '‐evalue 1e‐10 ‐num_alignments 1'. The bidirectional optimal genes were reserved as one versus one gene pair.

### Syntenic and subgenome evolution analysis

MUMmer (v3.5) (Delcher *et al*., 2003) was used to compare the allopolyploid *B. napus* and their diploid relatives *B. rapa* and *B. oleracea* keeping only 1‐on‐1 alignments (in order to enrich for orthologous segments) with a minimum length of 20 Kb. Syntenic gene pairs were identified and plotted by MCScan (Python version) (Tang *et al*., 2008) with the parameters of '‐‐cscore 0.99'. Synteny gene pairs were identified between the two subgenomes using MCScan (Python version) (Tang *et al*., 2008), and *Ks* values were calculated by a set of Python scripts (https//github.com/tanghaibao/bio‐pipeline/tree/master/synonymous_calculation). OrthoFinder (v2.2.6) (Emms and Kelly, 2015) was used to find the single‐copy genes in these genomes using salt cress (*Eutrema salsugineum v1.0,* download from Phytozome 12 https//phytozome.jgi.doe.gov/pz/portal.html#) as outgroup. For each ortho‐group, species phylogeny tree was reconstructed using RAxML (v8.2.12) (Figure S11b) (Stamatakis, 2014).

### SNP calling and annotation

The resequencing data of 991 *B. napus* accessions (658 winter, 145 semi‐winter, 188 spring) were downloaded from previous published paper (Wu *et al*., 2019a) and trimmed by Trimmomatic (v0.35) (Bolger *et al*., 2014). Then, the SNPs were called by GATK (v3.7) pipeline (McKenna *et al*., 2010) using ZS11_PB genome as reference and filtered with the following parameters QD < 1.5 || QUAL < 30.0 || DP < 5 || MQ0>= 4 && ((MQ0/ (1.0 * DP))> 0.1) ‐‐clusterWindowSize 10. Based on the annotation of ZS11_PB genome, the SNPs data sets identified above with missing rate less than 0.25 were annotated using SnpEff (v4.3m) (Cingolani *et al*., 2012).

### ML tree and the identification of selective sweeps

The SNPs in CDS regions with missing rate < 0.02 were reserved for constructing maximum‐likelihood (ML) tree by FastTree (v2.2.10) (Price *et al*., 2009), and the population structure was analysed used fastStructure (v1.0) (Raj *et al*., 2014). Additionally, selective sweeps were identified in whole genome using SNP data sets with missing rate less than 0.25. The population differentiation index (F_ST_) and nucleotide diversity (π) were calculated with 100 Kb windows and 10 Kb sliding steps through VCFtools (v0.1.13) (Danecek *et al*., 2011). Then, the genomic regions that contain sites with the highest 5% value of F_ST_ and the lowest or highest 5% of π ratio between ecotype groups were retained as the selective sweeps. The distribution of NLR genes and selective sweeps in Figure 5d was drawn by RIdeogram package (https//cran.r‐project.org/web/packages/RIdeogram/vignettes/RIdeogram.html).

## Conflict of interest

The authors declared that they have no conflict of interest to this work.

## Authors' contributions

L.Z., H.T. and S.L. conceived the study. X.C., L.Z., C.T., X. Z., A.S., M.H. and W.D. performed the analyses. X.C. and L.Z. wrote the manuscript. L.Z., C.T., F.C., Y.W., J.T., S.L and H.T. revised the manuscript and contributed to discussion. All authors read and approved the final manuscript.

## Supporting information


**Figure S1** Work flow of assembly in allotetraploid *B. napus* ZS11_PB.
**Figure S2** ZS11_PB pseudochromosome reconstructed from four published maps using ALLMAPS. DY, Z7, GP, TN with equal weights of 1.
**Figure S3** The Hi‐C intra‐chromosomal heatmap is displayed for ZS11_PB (a) and ZS11_NGS (b) based on HiCPlotter.
**Figure S4** The distribution of genes (a) and TEs (b) along chromosomes in ZS11_PB and ZS11_NGS.
**Figure S5** TE distribution and Kimura analysis of ZS11_NGS (a) and ZS11_PB (b).
**Figure S6** Distribution of the centromere unit length in ZS11_PB genome.
**Figure S7** The characteristics of recently amplified LTR‐RTs.
**Figure S8** The young LTR‐RTs specifically amplified in ZS11_PB genome.
**Figure S9** The distribution of TE‐related genes along chromosomes in ZS11_PB, ZS11_NGS and Darmor‐*bzh*.
**Figure S10** Macrosynteny plots of each chromosome among ZS11_PB, diploid parents and Darmor‐*bzh* (a).
**Figure S11** Evolutionary analysis among allopolyploid and their diploid parents.
**Figure S12** Top 20 of GO terms (Biological Process) and KEGG enrichment analysis in the A (a) and C (b) subgenome of ZS11_PB, respectively.
**Figure S15** Population structure analysis using SNPs within CDS region (with missingness < 2% of the genotyped accessions).
**Figure S16** KEGG pathway annotation in the selected regions between different ecotypes.

**Figure S13** Distribution of NLR genes (new annotated NLR genes marked by red font) on chromosomes.

**Figure S14** A maximum likelihood (ML) tree of NLR genes between ZS11_PB and known NLR genes of *A. thaliana* and *Glycine max*.

**Tables S1** Sequencing data used for the ZS11_PB genome assembly.
**Tables S2** Summary of the transcriptomes and their mapping rate on the ZS11_PB genome assembly.
**Tables S3** Statistics of the genome assembly.
**Tables S4** Distribution of the chromosome length and gene numbers.
**Tables S5** Assessment of ZS11_PB genome assemblies based on Illumina short reads.
**Tables S6** BUSCO analysis of different genomes.
**Tables S7** CEGMA analysis of genome assemblies.
**Tables S8** Statistics of mapping the full‐length transcriptome data to ZS11_PB genome assembly using GMAP.
**Tables S9** Summary of repeat annotation.
**Tables S10** Summary of the chromosome length and the centromeric repeats in ZS11_PB genome.
**Tables S11** The total number and length of different aged LTR‐RTs located in centromere regions.
**Tables S12** BUSCO analysis of transcriptome based on different RNA‐seq assembled strategies.
**Tables S13** The comparison of TE‐related genes between ZS11_PB, ZS11_NGS and Darmor‐*bzh*.
**Tables S14** Functional annotation of protein‐coding genes of ZS11_PB genome.
**Tables S15** Some of the ortholog genes between ZS11_PB and parent genome (*B. rapa* and *B. oleracea*).
**Tables S16** Top 20 of GO terms (Biological Process) and KEGG enrichment analysis in the A and C subgenome of ZS11_PB genome.
**Tables S17a** The NLR genes in *B. napus* cultivar 'ZS11_PB'.
**Tables S17b** Distribution of the NLR genes in ZS11_PB.
**Tables S18** List of the paired NLR genes in ZS11_PB genome.
**Tables S19** The summary of SNPs annotation using snpEff.
**Tables S20a** Summary of selective sweeps between the three ecotypes of *B. napus* (The top 1% of F_ST_).
**Tables S20b** The distribution of selective sweeps between the three ecotypes of *B. napus* based on ZS11_PB genome (The intersect with the top 5% of F_ST_ and π ratio).
**Tables S21a** Top 20 of KEGG enrichment analysis within selective regions between different ecotypes of *B. napus*.
**Tables S21b** The ortholog genes within selective regions between *A. thaliana* and ZS11_PB.
**Tables S22** The identified QTL regions against three major diseases in Darmor‐*bzh* and the corresponding interval in ZS11_PB.
**Tables S23** The selective sweep regions between semi‐winter and winter ecotypes.
**Tables S24** The selective sweep regions between semi‐winter and spring ecotypes.
**Tables S25** The selective sweep regions between spring and winter ecotypes.
**Tables S26** NLR genes in selective sweep regions.

## Data Availability

All raw PacBio Sequel and Hi‐C sequence reads of genome, RNA‐seq Illumina paired‐end reads and Iso‐Seq of transcriptome for the *B. napus* cultivar 'Zhongshuang 11' have been submitted to NCBI BioProject PRJNA640262 (see URLs). The genome assembly and annotation of ZS11_PB are available at National Genomics Data Center (GWH accession number GWHANRE00000000).
