# Supplementary material for: A high‐quality Brassica napus genome reveals expansion of transposable elements, subgenome evolution and disease resistance
Source: Plant Biotechnol J. 2020 Nov 20;19(3):615–30. doi: 10.1111/pbi.13493 (PMC7955885; doi:10.1111/pbi.13493)
Supplement: Supplementary file 1 — Figure S1 Work flow of assembly in allotetraploid B. napus ZS11_PB. Figure S2 ZS11_PB pseudochromosome reconstructed from four published maps using ALLMAPS. DY, Z7, GP, TN with equal weights of 1. Figure S3 The Hi‐C intra‐chromosomal heatmap is displayed for ZS11_PB (a) and ZS11_NGS (b) based on HiCPlotter. Figure S4 The distribution of genes (a) and TEs (b) along chromosomes in ZS11_PB and ZS11_NGS. Figure S5 TE distribution and Kimura analysis of ZS11_NGS (a) and ZS11_PB (b). Figure S6 Distribution of the centromere unit length in ZS11_PB genome. Figure S7 The characteristics of recently amplified LTR‐RTs. Figure S8 The young LTR‐RTs specifically amplified in ZS11_PB genome. Figure S9 The distribution of TE‐related genes along chromosomes in ZS11_PB, ZS11_NGS and Darmor‐bzh. Figure S10 Macrosynteny plots of each chromosome among ZS11_PB, diploid parents and Darmor‐bzh (a). Figure S11 Evolutionary analysis among allopolyploid and their diploid parents. Figure S12 Top 20 of GO terms (Biological Process) and KEGG enrichment analysis in the A (a) and C (b) subgenome of ZS11_PB, respectively. Figure S15 Population structure analysis using SNPs within CDS region (with missingness < 2% of the genotyped accessions). Figure S16 KEGG pathway annotation in the selected regions between different ecotypes. [file PBI-19-615-s001.pdf]

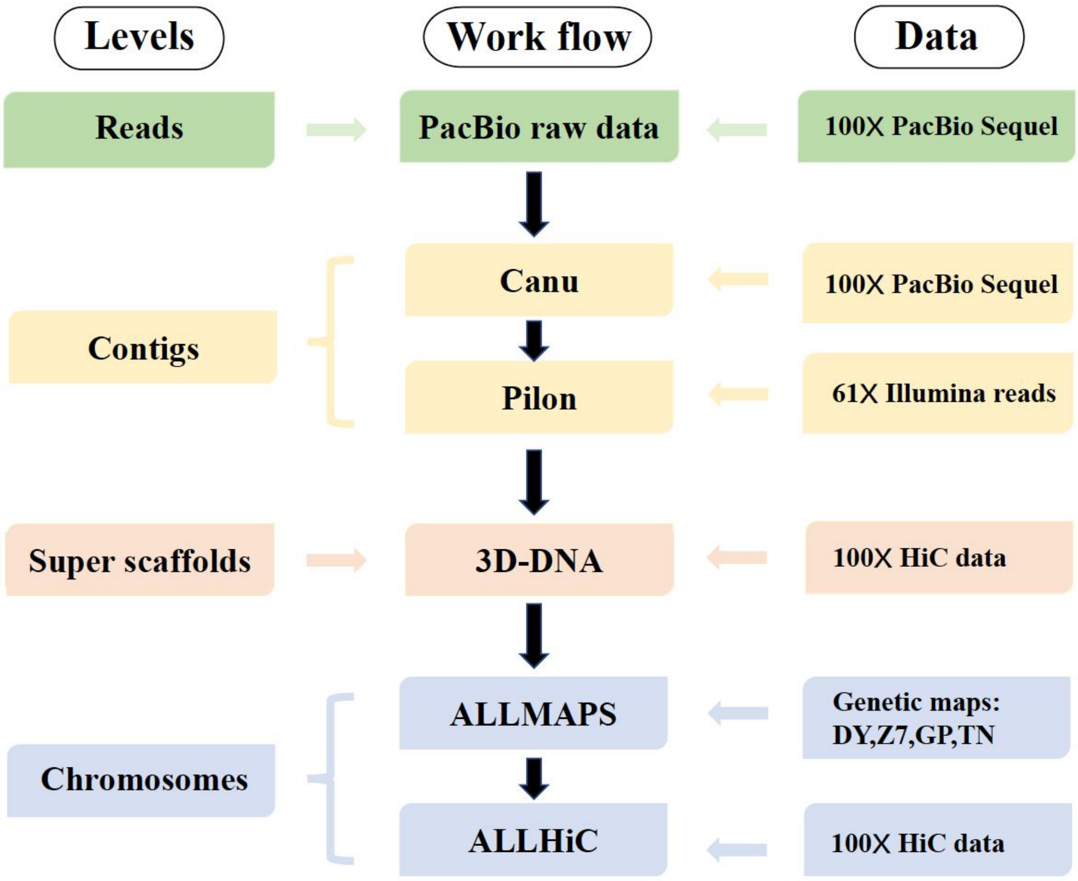

Figure S1: **Work flow of assembly in allotetraploid *B. napus* ZS11_PB.** Contigs were assembled using 100× PacBio data and polished by 61× Illumina reads, followed by Hi-C based scaffolding and chromosome anchoring based on linkage maps.

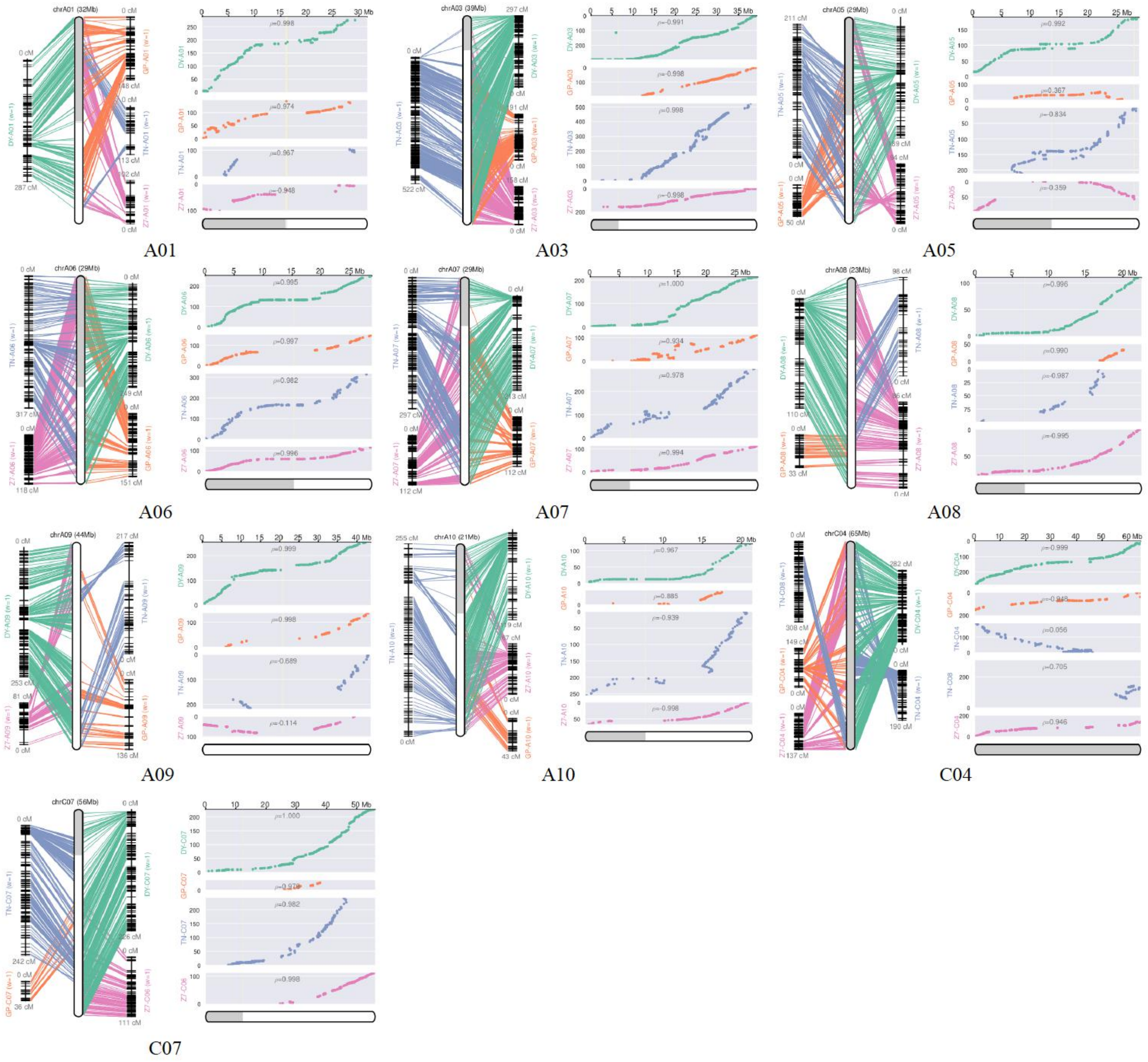

Figure S2: ZS11_PB pseudochromosome reconstructed from four published maps using ALLMAPS. DY, Z7, GP, TN with equal weights of 1. In each picture, the left panels represent the connections between the reconstructed chromosome and the map positions. The right panels are scatter plot which the dots represent the physical locations (x axis) on the chromosome versus the map locations (y axis) of markers. Rho (ρ) means the Pearson correlation coefficient between -1 to 1. Different colors mean different maps. Some chromosomes were not showed here means that one supper-scaffold denoted as one chromosome.

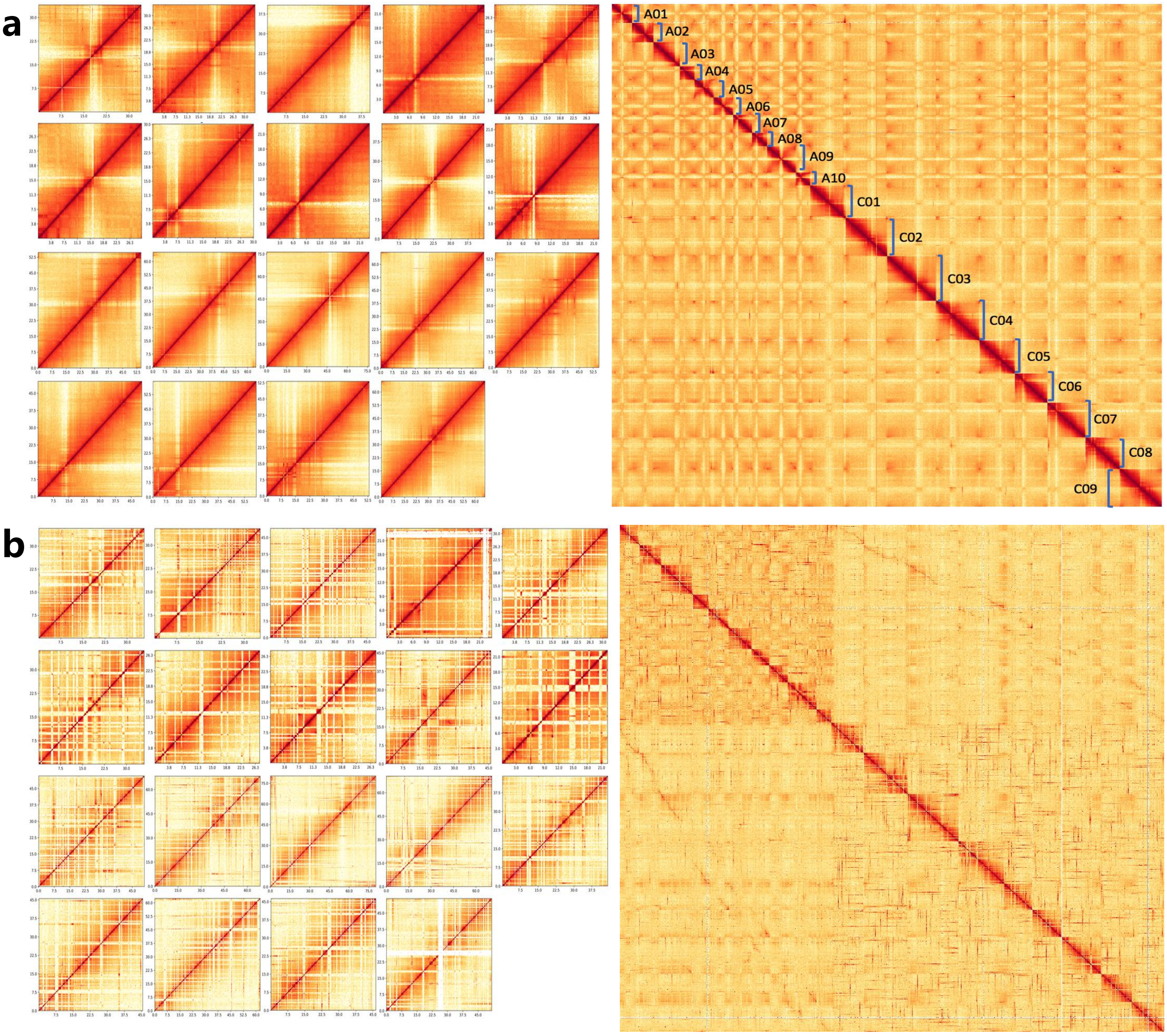

Figure S3: The Hi-C intra-chromosomal heatmap is displayed for ZS11_PB (a) and ZS11_NGS (b) based on HiCPlotter. The left panels represent the heatmap of each chromosome with 150 kb resolution from chromosome A01 to C09. The right panels represent the whole genome Hi-C contact map with 500 kb resolution. Darker red pixel means a higher interaction links.

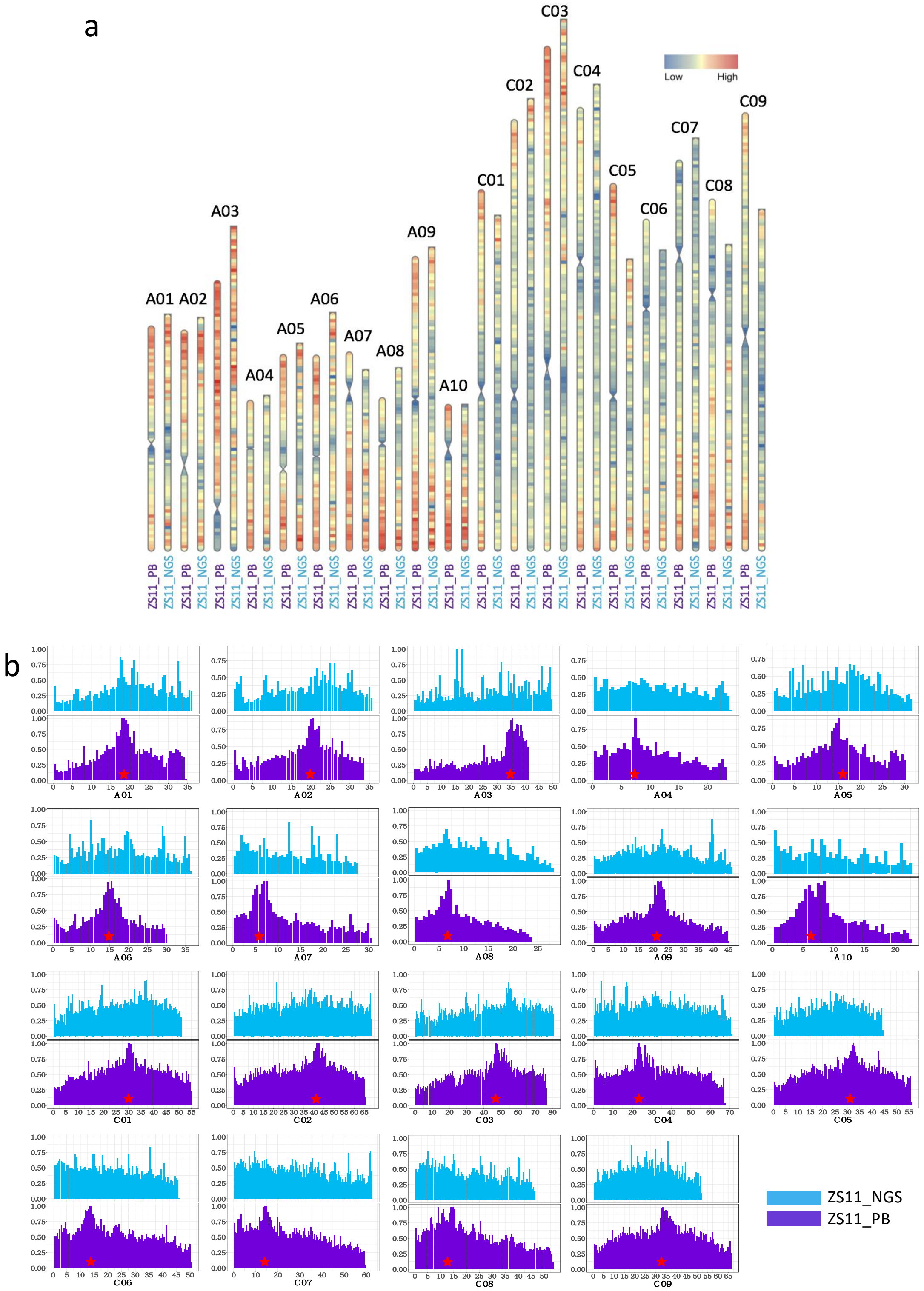

Figure S4: The distribution of genes (a) and TEs (b) along chromosomes in ZS11_PB and ZS11_NGS. (a) The density of gene along chromosomes in ZS11_PB and ZS11_NGS. (b) The distribution of TEs along chromosomes in ZS11_PB and ZS11_NGS. The *x* axis represents the chromosome position (Mb). The *y* axis represents the rate of TEs length within 500 Kb window size.

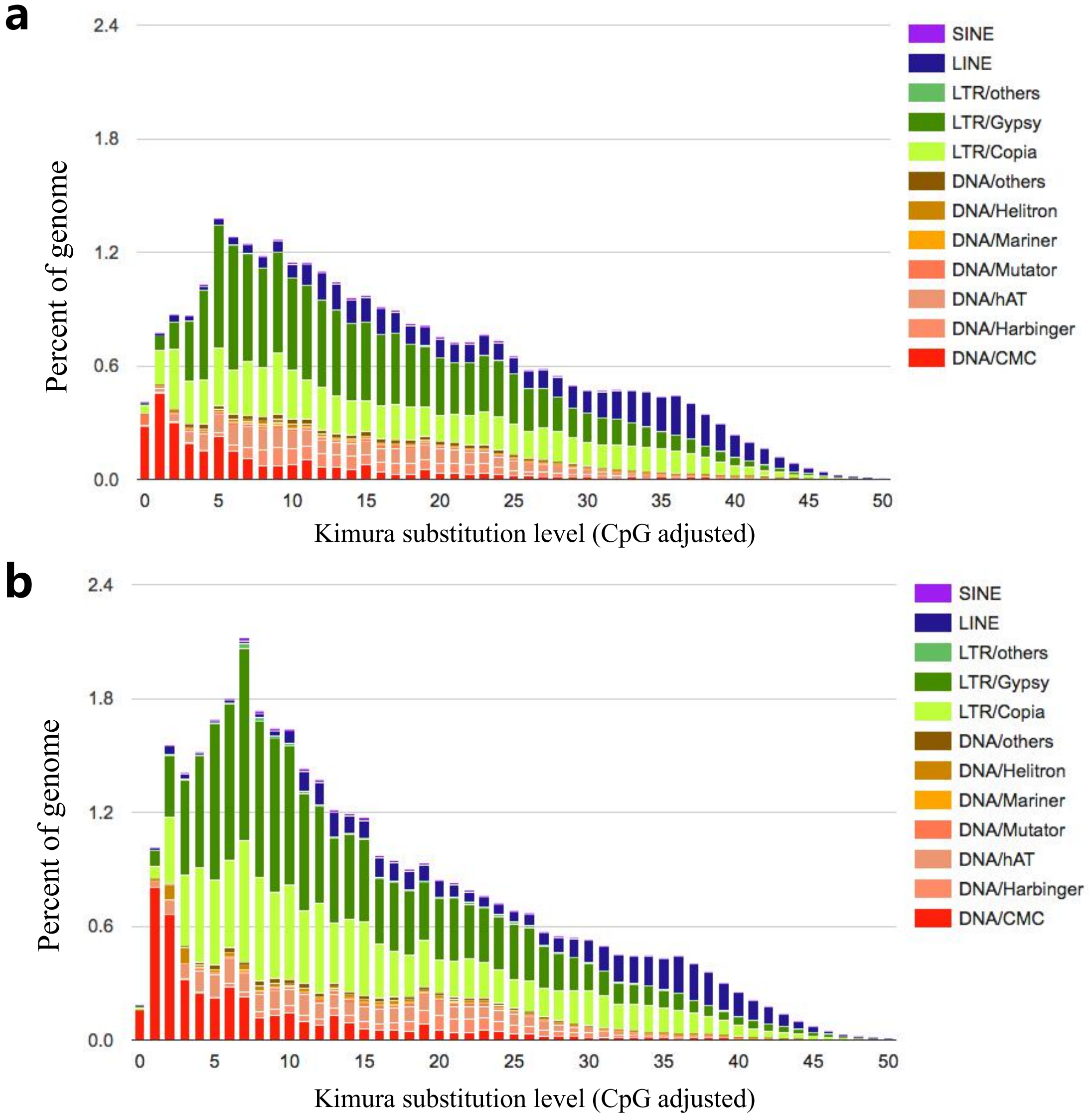

Figure S5: TE distribution and Kimura analysis of ZS11_NGS (a) and ZS11_PB (b).

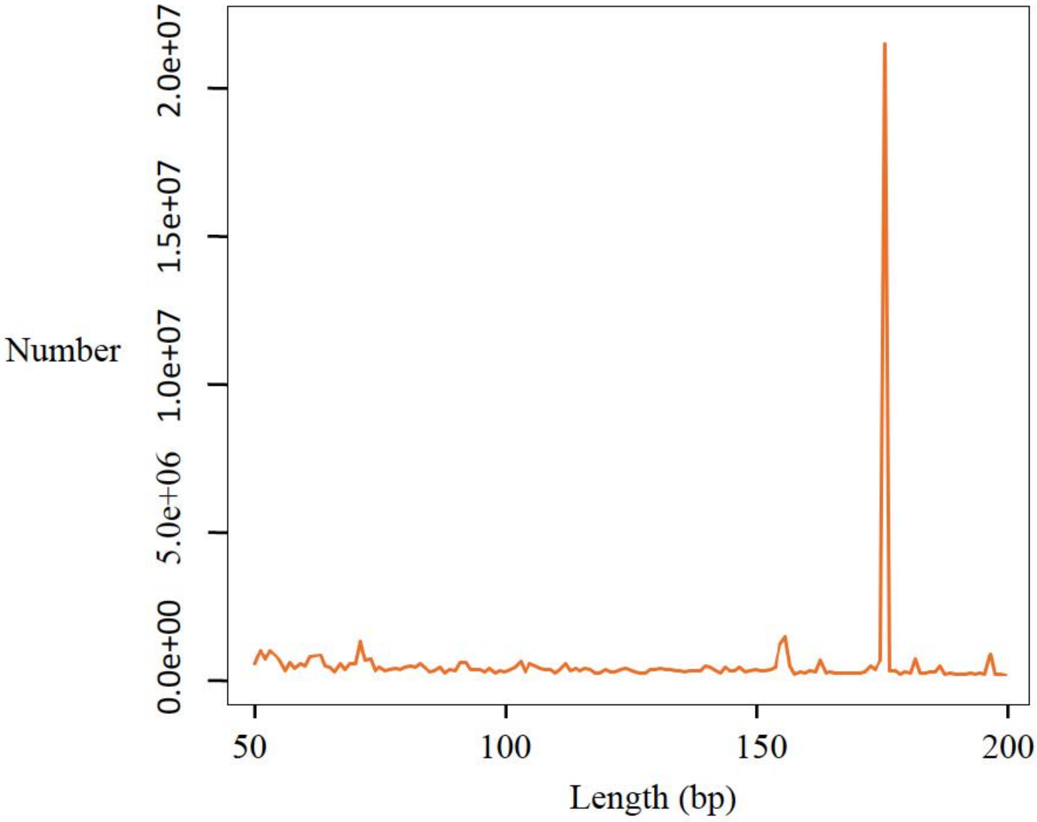

Figure S6: Distribution of the centromere unit length in ZS11_PB genome. The *x* and *y* axis represent the length and the total number of a unit centromere, respectively. The highest point is 176 bp.

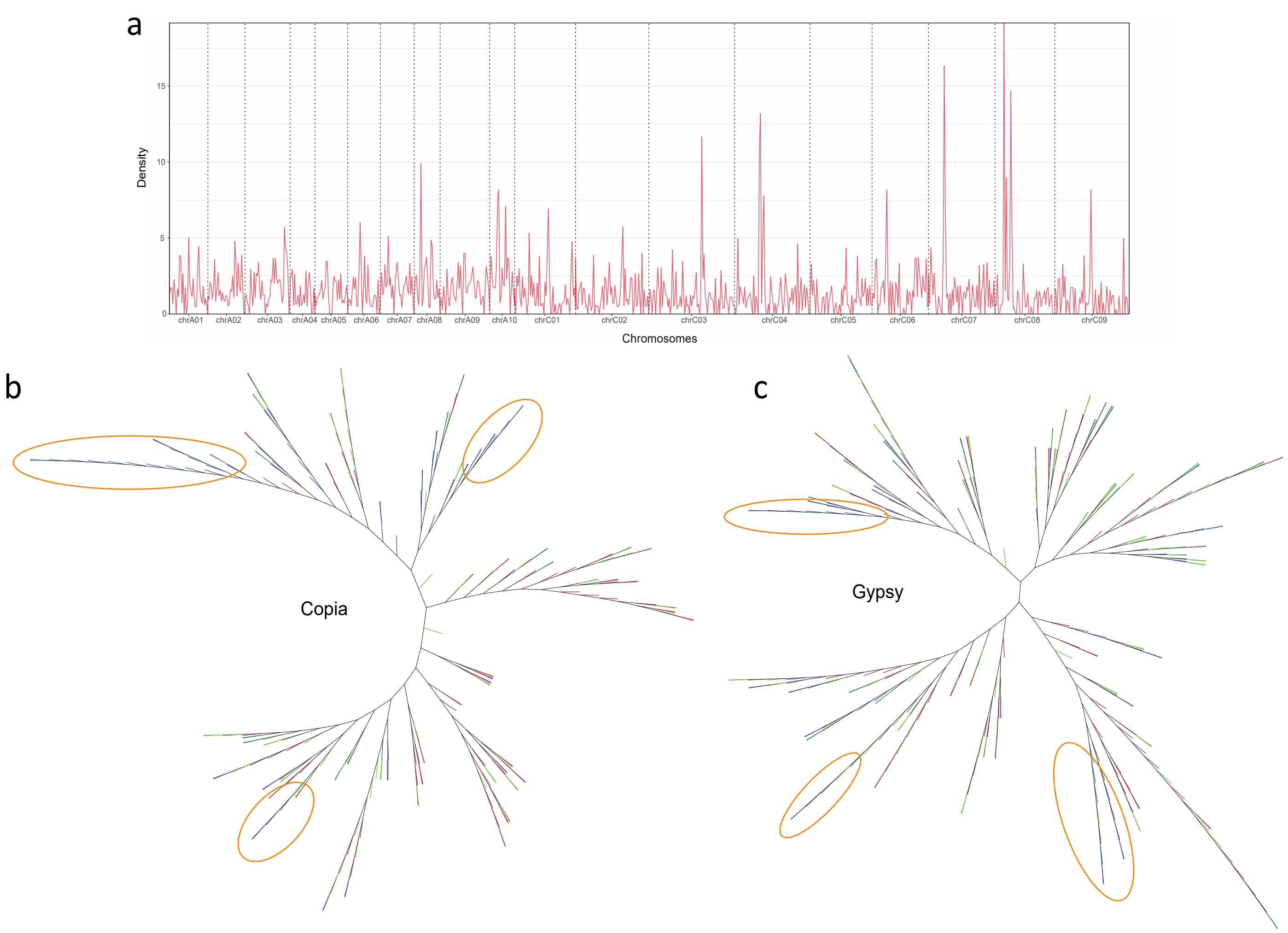

Figure S7: The characteristics of recently amplified LTR-RTs. (a) The chromosomal distribution of LTR-RTs with age < 0.2 My (million years) in *B. napus*. The density represented the percentage of total LTR-RTs size (bp) in each 1 Mb genomic region. (b) Phylogeny of the *Copia*-like elements. (c) Phylogeny of the *Gypsy*-like elements. The blue, green and red clades represented the LTR-RTs with age < 0.2 My, 0.2-1 My, and > 1 My. The HMMER (v3.3) (Eddy, 2011) was used to predict the RT domains by scanning Pfam database (version 26.0). The MUSCLE (v3.8.31) (Edgar, 2004) was employed to align the RT protein sequences. MEGA software (Tamura et al., 2007) were used to construct the maximum likelihood (ML) trees.

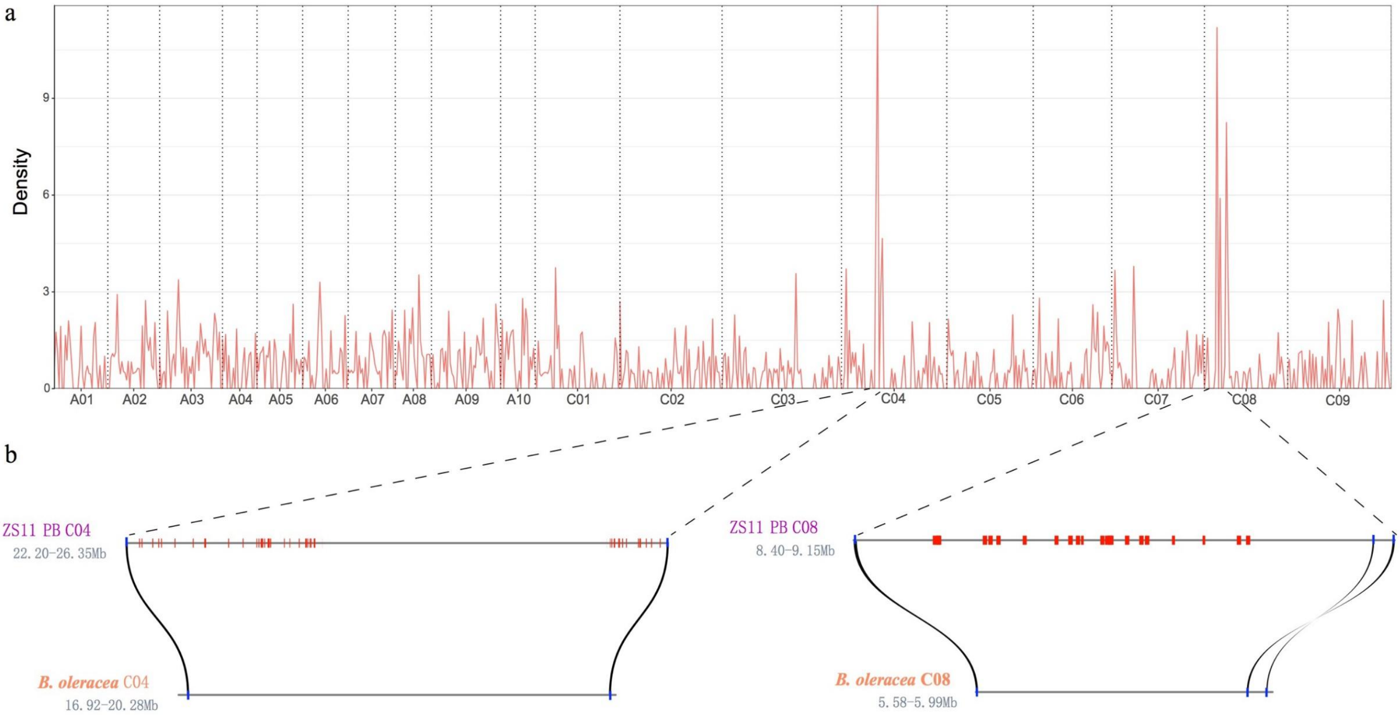

Figure S8: The young LTR-RTs specifically amplified in ZS11_PB genome. (a) the chromosomal distribution of LTR-RTs with age < 10,000 in *B. napus*. The density represented the percentage of total LTR-RTs size (bp) in each 1 Mb genomic region. (b) Two genomic regions in C04 and C08 showed clustered LTR-RT amplified in *B. napus* but not presented in syntenic regions of *B. oleracea*. The red boxes represent LTR-RTs, and the black curves represent the collinear genes.

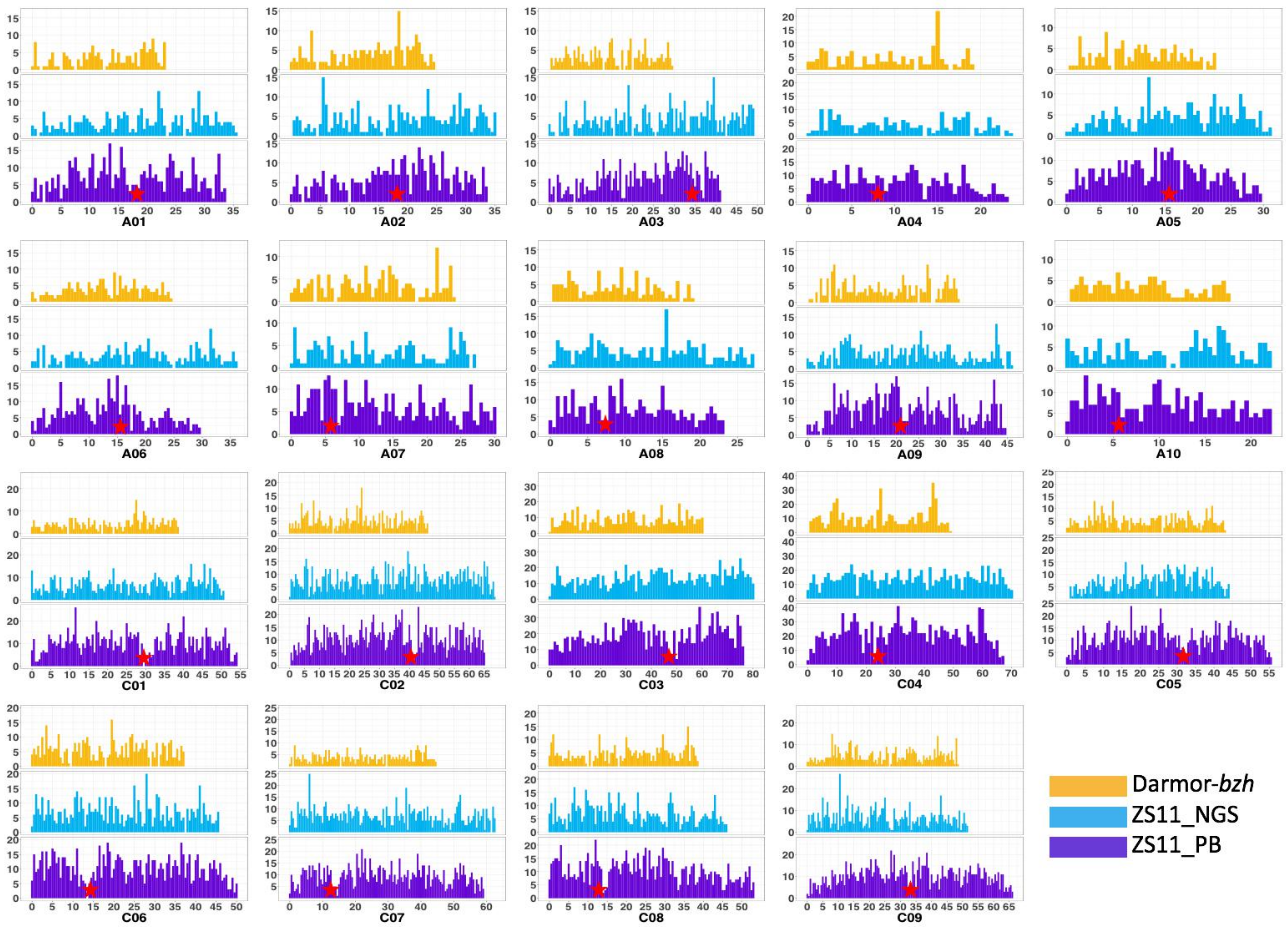

Figure S9: The distribution of TE-related genes along chromosomes in ZS11_PB, ZS11_NGS and Darmor-*bzh*. The *x* and *y* axis represent the chromosome position (Mb) and TE-related gene number with 500 Kb window size. The red pentacle is the position of the centromeres in ZS11_PB.

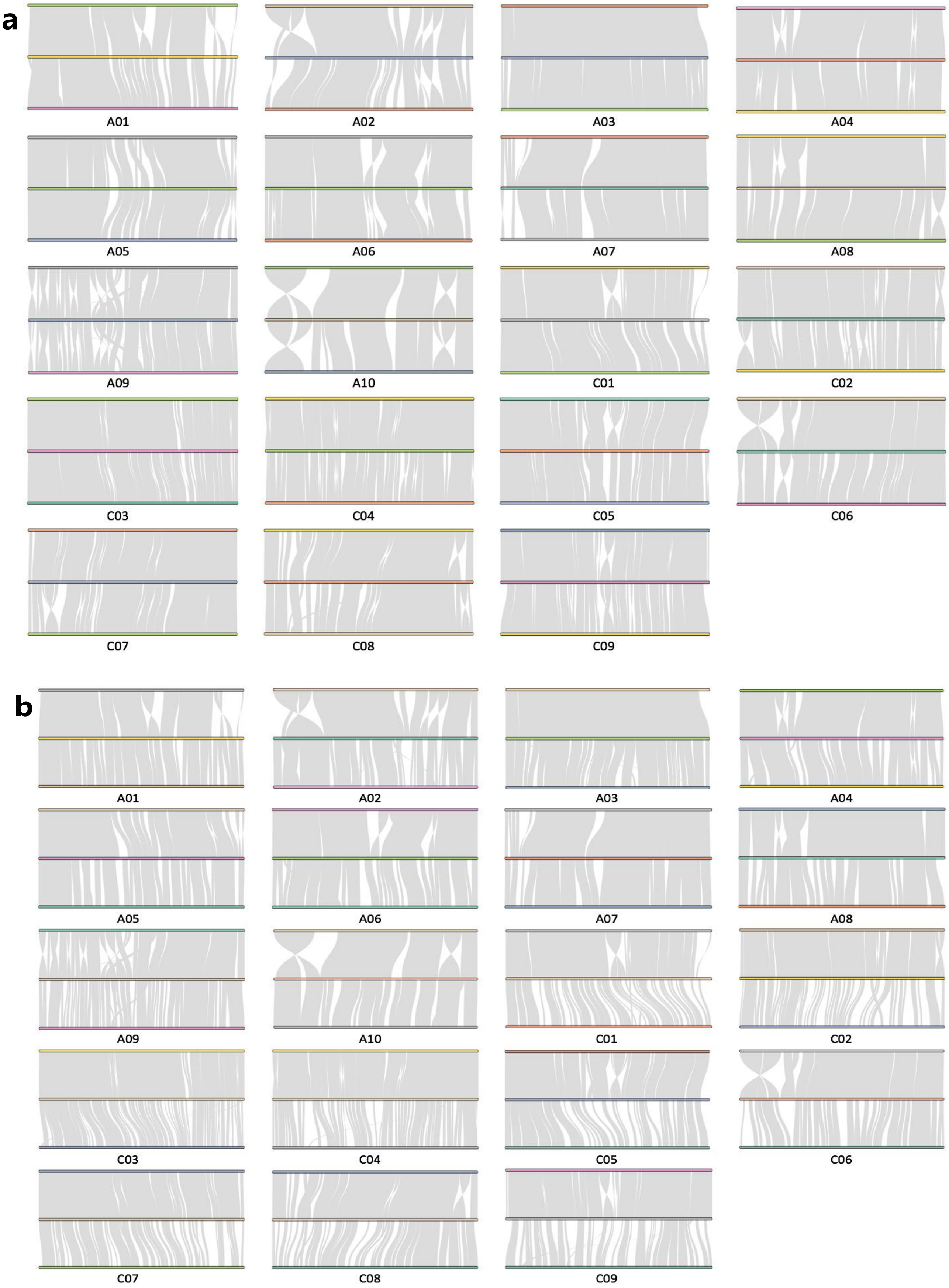

Figure S10: Macrosynteny plots of each chromosome among ZS11_PB, diploid parents and Darmor-*bzh* (a). ZS11_PB, diploid parents and ZS11_NGS (b). Grey areas represent the synteny blocks.

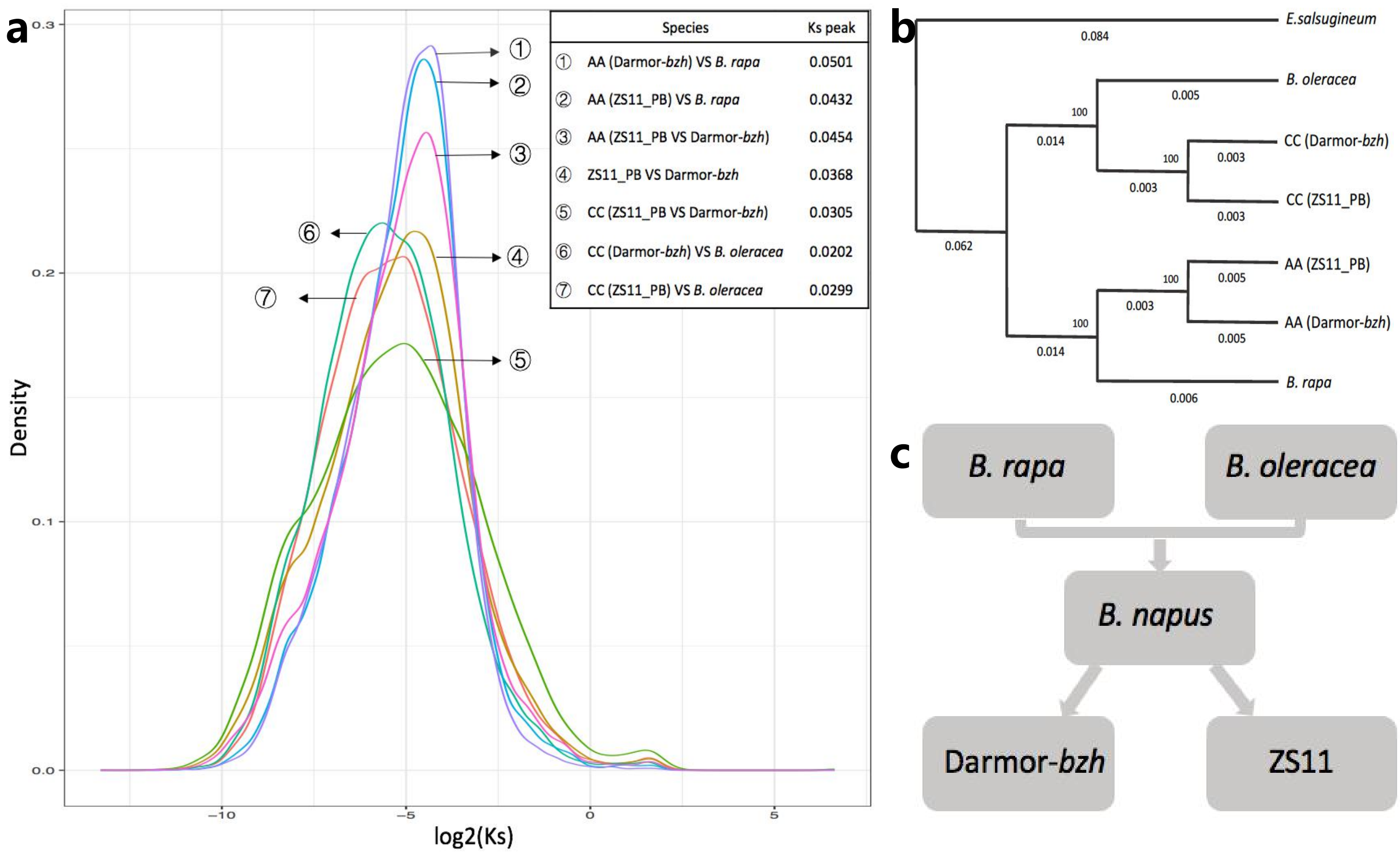

Figure S11: Evolutionary analysis among allopolyploid and their diploid parents. (a) *Ks* distribution of the gene pairs between selected genomes: diploid progenitors, *B. rapa* and *B. oleracea*, ZS11_PB and Darmor-*bzh*. AA or CC: the subgenome of the allopolyploid within ZS11_PB. (b) Phylogenetic tree with *E. salsugineum* as outgroup. The value below the branch represents the evolutionary distance (units: substitutions/site), while the above value represents the supported degree. (c) Inferred evolution model of *B. napus*.

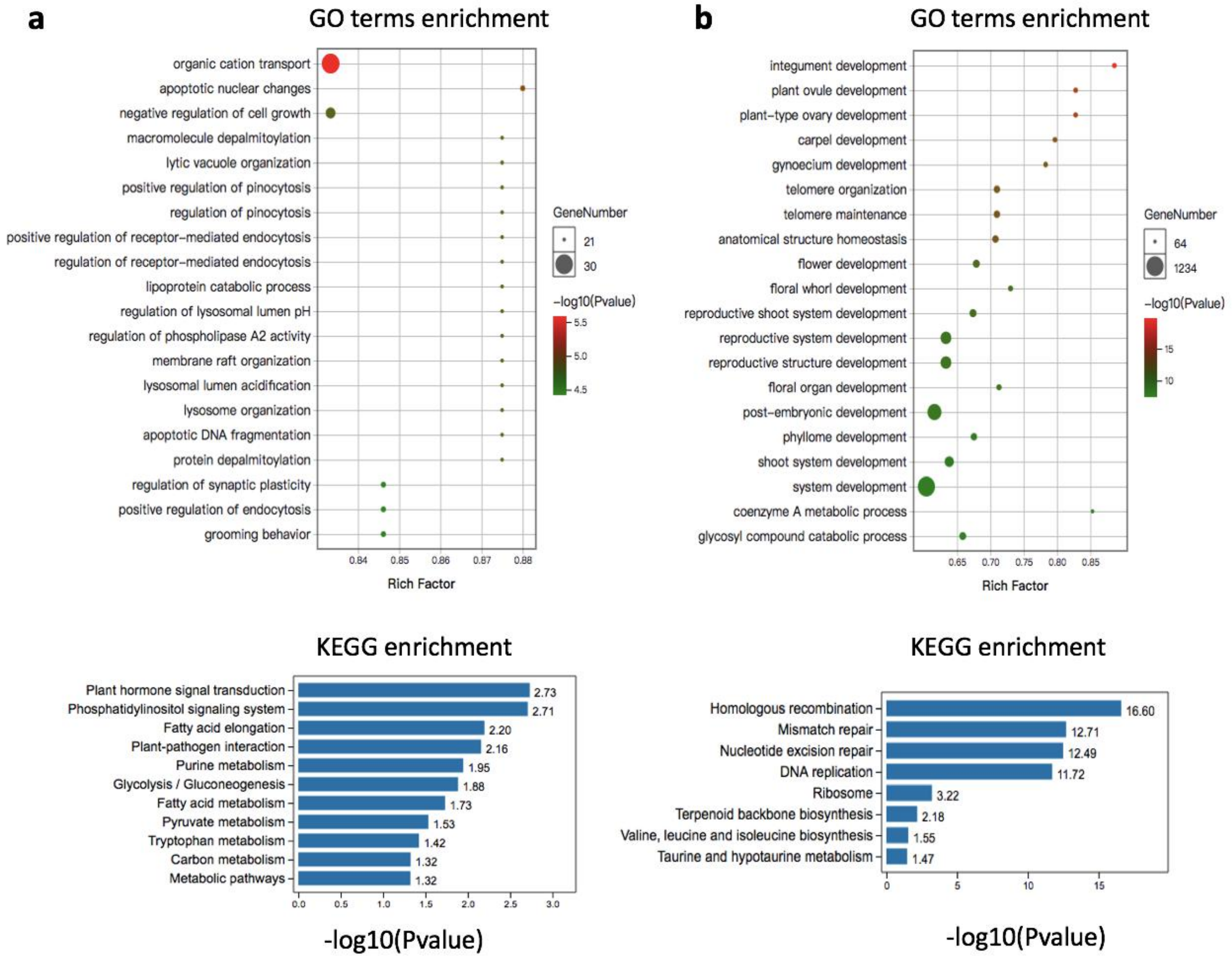

Figure S12: Top 20 of GO terms (Biological Process) and KEGG enrichment analysis in A (a) and C (b) subgenome of ZS11_PB, respectively. Both GO and KEGG analysis were performed at online website (www.omicshare.com/tools). A detailed list of genes was shown in the Supplementary Table S16.

Figure S13: Distribution of NLR genes (new annotated NLR genes marked by red font) on chromosomes. This figure draw by online website MG2C v2.1 (http://mg2c.iask.in/mg2c_v2.1/). (This figure is in a separate PDF file.)

Figure S14: A maximum likehood (ML) tree of NLR genes between ZS11_PB and known NLR genes of *A. thaliana* and *Glycine max*. The known R genes were downloaded from Phytozome 12.1 (https://phytozome.jgi.doe.gov/pz/portal.html#). (This figure is in a separate PDF file.)

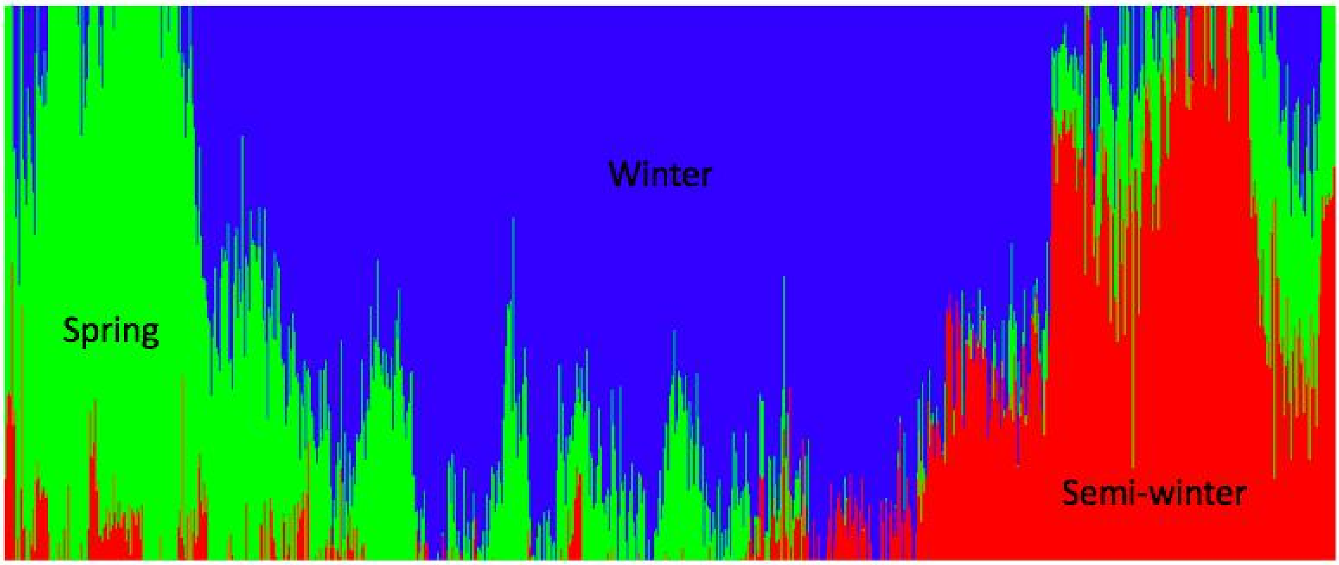

Figure S15: Population structure analysis using SNPs within CDS region (with missingness < 2% of the genotyped accessions).

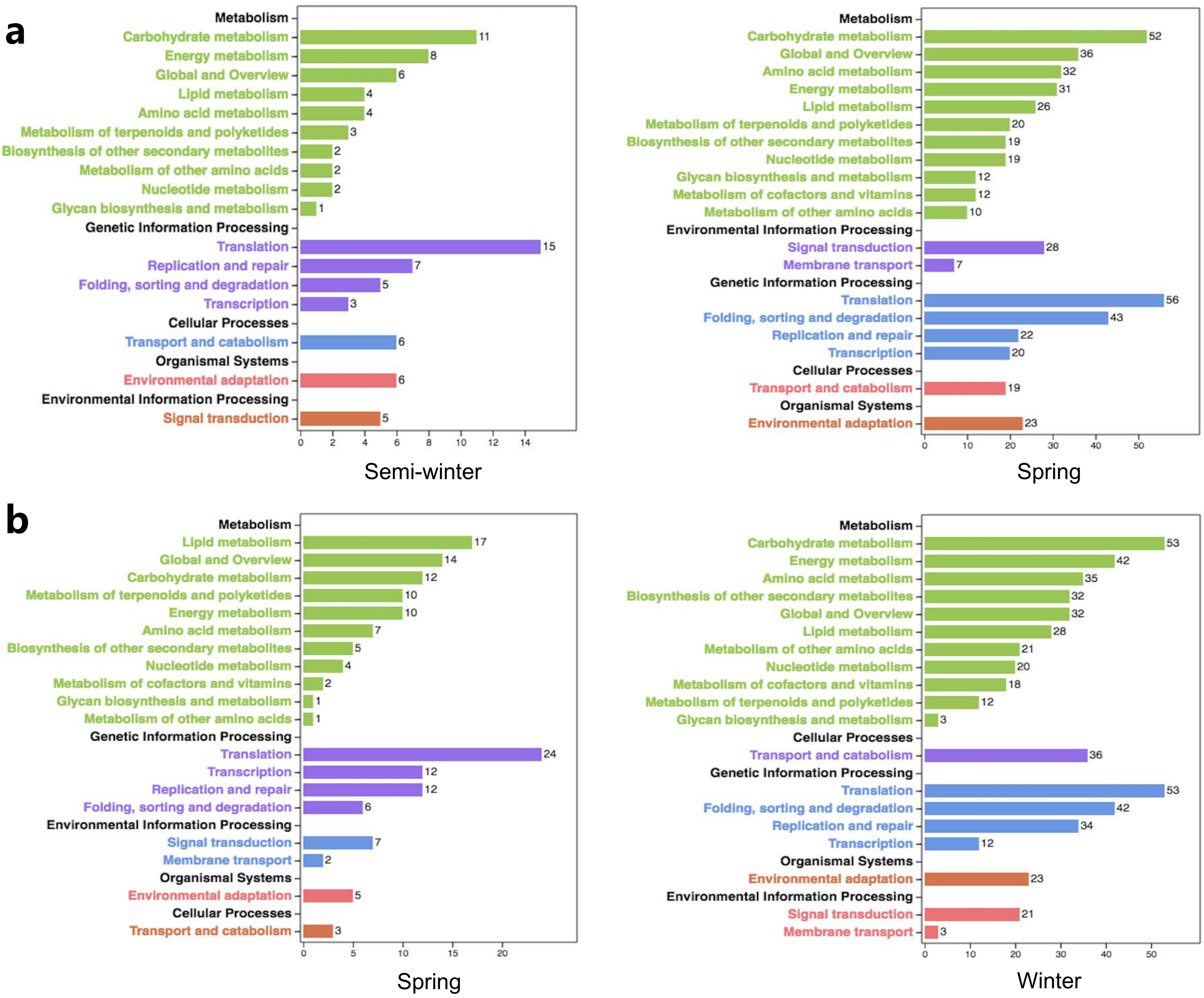

Figure S16: KEGG pathway annotation in the selected regions between different ecotypes. (a) KEGG pathway annotation in the selected regions between semi-winter and spring ecotype groups. (b) KEGG pathway annotation in the selected regions between spring and winter ecotype groups. The black font on the *y*-axis represents KEGG B class, the color font represents the specific pathway under the class, and the *x*-axis represents the number of genes in the pathway. A detailed list of genes was shown in the Supplementary Table S21a.

Eddy, S.R. (2011) Accelerated Profile HMM Searches. *PLoS Comput Biol* **7**, e1002195.

Edgar, R.C. (2004) MUSCLE: multiple sequence alignment with high accuracy and high throughput. *Nucleic Acids Res* **32**, 1792-1797.

Tamura, K., Dudley, J., Nei, M. and Kumar, S. (2007) MEGA4: Molecular Evolutionary Genetics Analysis (MEGA) software version 4.0. *Mol Biol Evol* **24**, 1596-1599.
