# Supplementary material for: A high‐quality Brassica napus genome reveals expansion of transposable elements, subgenome evolution and disease resistance
Source: Plant Biotechnol J. 2020 Nov 20;19(3):615–30. doi: 10.1111/pbi.13493 (PMC7955885; doi:10.1111/pbi.13493)
Supplement: Supplementary file 2 — Figure S13 Distribution of NLR genes (new annotated NLR genes marked by red font) on chromosomes. [file PBI-19-615-s002.pdf]

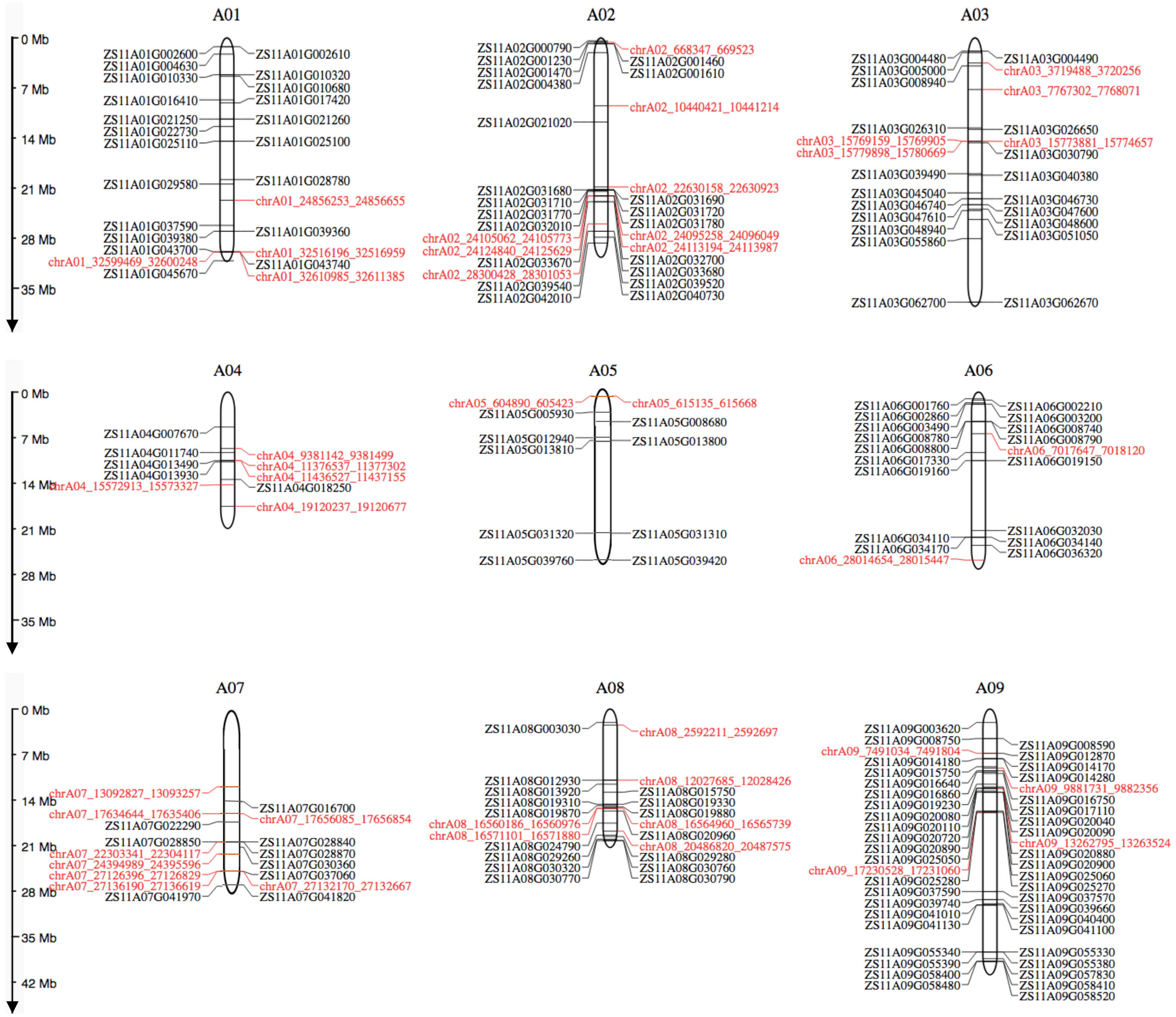

A01
A02
A03
A04
A05
A06
A07
A08
A09
0 Mb
7 Mb
14 Mb
21 Mb
28 Mb
35 Mb
42 Mb
ZS11A01G002600
ZS11A01G004630
ZS11A01G010330
ZS11A01G016410
ZS11A01G021250
ZS11A01G022730
ZS11A01G025110
ZS11A01G029580
ZS11A01G037590
ZS11A01G039380
ZS11A01G043700
chrA01_32599469_32600248
ZS11A01G045670
ZS11A01G002610
ZS11A01G010320
ZS11A01G010680
ZS11A01G017420
ZS11A01G021260
ZS11A01G025100
ZS11A01G028780
chrA01_24856253_24856655
ZS11A01G039360
chrA01_32516196_32516959
ZS11A01G043740
chrA01_32610985_32611385
ZS11A02G000790
ZS11A02G001230
ZS11A02G001470
ZS11A02G004380
ZS11A02G021020
ZS11A02G031680
ZS11A02G031710
ZS11A02G031770
ZS11A02G032010
chrA02_24105062_24105773
chrA02_24124840_24125629
ZS11A02G033670
chrA02_28300428_28301053
ZS11A02G039540
ZS11A02G042010
chrA02_668347_669523
ZS11A02G001460
ZS11A02G001610
chrA02_10440421_10441214
chrA02_22630158_22630923
ZS11A02G031690
ZS11A02G031720
ZS11A02G031780
chrA02_24095258_24096049
chrA02_24113194_24113987
ZS11A02G032700
ZS11A02G033680
ZS11A02G039520
ZS11A02G040730
ZS11A03G004480
ZS11A03G005000
ZS11A03G008940
ZS11A03G026310
chrA03_15769159_15769905
chrA03_15779898_15780669
ZS11A03G039490
ZS11A03G045040
ZS11A03G046740
ZS11A03G047610
ZS11A03G048940
ZS11A03G055860
ZS11A03G062700
ZS11A03G004490
chrA03_3719488_3720256
chrA03_7767302_7768071
ZS11A03G026650
chrA03_15773881_15774657
ZS11A03G030790
ZS11A03G040380
ZS11A03G046730
ZS11A03G047600
ZS11A03G048600
ZS11A03G051050
ZS11A03G062670
ZS11A04G007670
ZS11A04G011740
ZS11A04G013490
ZS11A04G013930
chrA04_15572913_15573327
chrA04_9381142_9381499
chrA04_11376537_11377302
chrA04_11436527_11437155
ZS11A04G018250
chrA04_19120237_19120677
chrA05_604890_605423
ZS11A05G005930
ZS11A05G012940
ZS11A05G013810
ZS11A05G031320
ZS11A05G039760
chrA05_615135_615668
ZS11A05G008680
ZS11A05G013800
ZS11A05G031310
ZS11A05G039420
ZS11A06G001760
ZS11A06G002860
ZS11A06G003490
ZS11A06G008780
ZS11A06G008800
ZS11A06G017330
ZS11A06G019160
ZS11A06G034110
ZS11A06G034170
chrA06_28014654_28015447
ZS11A06G002210
ZS11A06G003200
ZS11A06G008740
ZS11A06G008790
chrA06_7017647_7018120
ZS11A06G019150
ZS11A06G032030
ZS11A06G034140
ZS11A06G036320
chrA07_13092827_13093257
chrA07_17634644_17635406
ZS11A07G022290
ZS11A07G028850
chrA07_22303341_22304117
chrA07_24394989_24395596
chrA07_27126396_27126829
chrA07_27136190_27136619
ZS11A07G041970
ZS11A07G016700
chrA07_17656085_17656854
ZS11A07G028840
ZS11A07G028870
ZS11A07G030360
ZS11A07G037060
chrA07_27132170_27132667
ZS11A07G041820
ZS11A08G003030
ZS11A08G012930
ZS11A08G013920
ZS11A08G019310
ZS11A08G019870
chrA08_16560186_16560976
chrA08_16571101_16571880
ZS11A08G024790
ZS11A08G029260
ZS11A08G030320
ZS11A08G030770
chrA08_2592211_2592697
chrA08_12027685_12028426
ZS11A08G015750
ZS11A08G019330
ZS11A08G019880
chrA08_16564960_16565739
ZS11A08G020960
chrA08_20486820_20487575
ZS11A08G029280
ZS11A08G030760
ZS11A08G030790
ZS11A09G003620
ZS11A09G008750
chrA09_7491034_7491804
ZS11A09G014180
ZS11A09G015750
ZS11A09G016640
ZS11A09G016860
ZS11A09G019230
ZS11A09G020080
ZS11A09G020110
ZS11A09G020720
ZS11A09G020890
ZS11A09G025050
chrA09_17230528_17231060
ZS11A09G025280
ZS11A09G037590
ZS11A09G039740
ZS11A09G041010
ZS11A09G041130
ZS11A09G055340
ZS11A09G055390
ZS11A09G058400
ZS11A09G058480
ZS11A09G008590
ZS11A09G012870
ZS11A09G014170
ZS11A09G014280
chrA09_9881731_9882356
ZS11A09G016750
ZS11A09G017110
ZS11A09G020040
ZS11A09G020090
chrA09_13262795_13263524
ZS11A09G020880
ZS11A09G020900
ZS11A09G025060
ZS11A09G025270
ZS11A09G037570
ZS11A09G039660
ZS11A09G040400
ZS11A09G041100
ZS11A09G055330
ZS11A09G055380
ZS11A09G057830
ZS11A09G058410
ZS11A09G058520

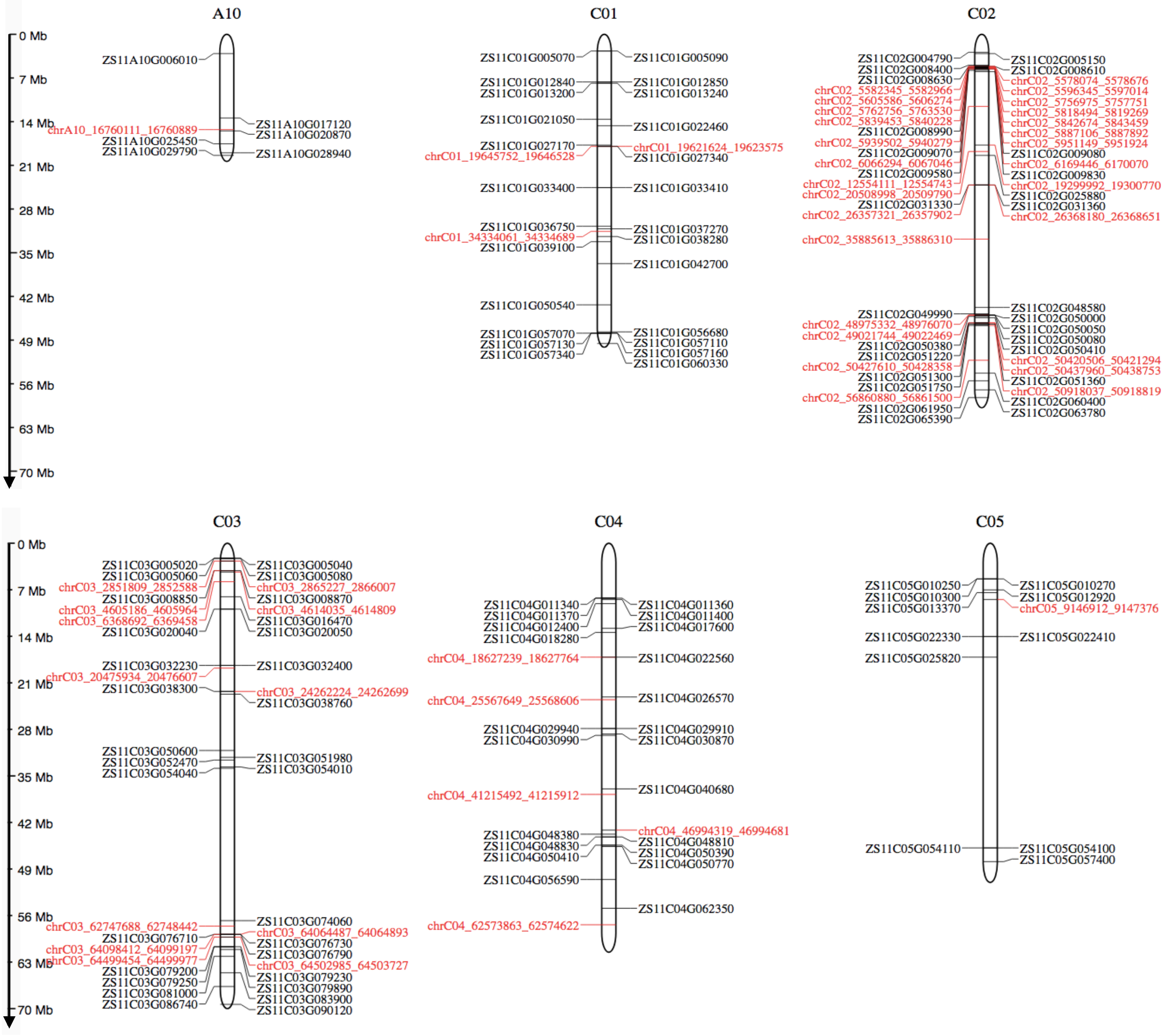
A10
C01
C02
C03
C04
C05
0 Mb
7 Mb
14 Mb
21 Mb
28 Mb
35 Mb
42 Mb
49 Mb
56 Mb
63 Mb
70 Mb
ZS11A10G006010
ZS11A10G017120
chrA10_16760111_16760889
ZS11A10G020870
ZS11A10G025450
ZS11A10G029790
ZS11A10G028940
ZS11C01G005070
ZS11C01G005090
ZS11C01G012840
ZS11C01G012850
ZS11C01G013200
ZS11C01G013240
ZS11C01G021050
ZS11C01G022460
ZS11C01G027170
chrC01_19621624_19623575
chrC01_19645752_19646528
ZS11C01G027340
ZS11C01G033400
ZS11C01G033410
ZS11C01G036750
ZS11C01G037270
chrC01_34334061_34334689
ZS11C01G038280
ZS11C01G039100
ZS11C01G042700
ZS11C01G050540
ZS11C01G057070
ZS11C01G056680
ZS11C01G057130
ZS11C01G057110
ZS11C01G057340
ZS11C01G057160
ZS11C01G060330
ZS11C02G004790
ZS11C02G005150
ZS11C02G008400
ZS11C02G008610
ZS11C02G008630
chrC02_5578074_5578676
chrC02_5582345_5582966
chrC02_5596345_5597014
chrC02_5605586_5606274
chrC02_5756975_5757751
chrC02_5762756_5763530
chrC02_5818494_5819269
chrC02_5839453_5840228
chrC02_5842674_5843459
ZS11C02G008990
chrC02_5887106_5887892
chrC02_5939502_5940279
chrC02_5951149_5951924
ZS11C02G009070
ZS11C02G009080
chrC02_6066294_6067046
chrC02_6169446_6170070
ZS11C02G009580
ZS11C02G009830
chrC02_12554111_12554743
chrC02_19299992_19300770
chrC02_20508998_20509790
ZS11C02G025880
ZS11C02G031330
ZS11C02G031360
chrC02_26357321_26357902
chrC02_26368180_26368651
chrC02_35885613_35886310
ZS11C02G048580
ZS11C02G049990
ZS11C02G050000
chrC02_48975332_48976070
ZS11C02G050050
chrC02_49021744_49022469
ZS11C02G050080
ZS11C02G050380
ZS11C02G050410
ZS11C02G051220
chrC02_50420506_50421294
chrC02_50427610_50428358
chrC02_50437960_50438753
ZS11C02G051300
ZS11C02G051360
ZS11C02G051750
chrC02_50918037_50918819
chrC02_56860880_56861500
ZS11C02G060400
ZS11C02G061950
ZS11C02G063780
ZS11C02G065390
ZS11C03G005020
ZS11C03G005040
ZS11C03G005060
ZS11C03G005080
chrC03_2851809_2852588
chrC03_2865227_2866007
ZS11C03G008850
ZS11C03G008870
chrC03_4605186_4605964
chrC03_4614035_4614809
chrC03_6368692_6369458
ZS11C03G016470
ZS11C03G020040
ZS11C03G020050
ZS11C03G032230
ZS11C03G032400
chrC03_20475934_20476607
ZS11C03G038300
chrC03_24262224_24262699
ZS11C03G038760
ZS11C03G050600
ZS11C03G051980
ZS11C03G052470
ZS11C03G054010
ZS11C03G054040
ZS11C03G074060
chrC03_62747688_62748442
chrC03_64064487_64064893
ZS11C03G076710
ZS11C03G076730
chrC03_64098412_64099197
ZS11C03G076790
chrC03_64499454_64499977
chrC03_64502985_64503727
ZS11C03G079200
ZS11C03G079230
ZS11C03G079250
ZS11C03G079890
ZS11C03G081000
ZS11C03G083900
ZS11C03G086740
ZS11C03G090120
ZS11C04G011340
ZS11C04G011360
ZS11C04G011370
ZS11C04G011400
ZS11C04G012400
ZS11C04G017600
ZS11C04G018280
chrC04_18627239_18627764
ZS11C04G022560
chrC04_25567649_25568606
ZS11C04G026570
ZS11C04G029940
ZS11C04G029910
ZS11C04G030990
ZS11C04G030870
ZS11C04G040680
chrC04_41215492_41215912
chrC04_46994319_46994681
ZS11C04G048380
ZS11C04G048810
ZS11C04G048830
ZS11C04G050390
ZS11C04G050410
ZS11C04G050770
ZS11C04G056590
ZS11C04G062350
chrC04_62573863_62574622
ZS11C05G010250
ZS11C05G010270
ZS11C05G010300
ZS11C05G012920
ZS11C05G013370
chrC05_9146912_9147376
ZS11C05G022330
ZS11C05G022410
ZS11C05G025820
ZS11C05G054110
ZS11C05G054100
ZS11C05G057400

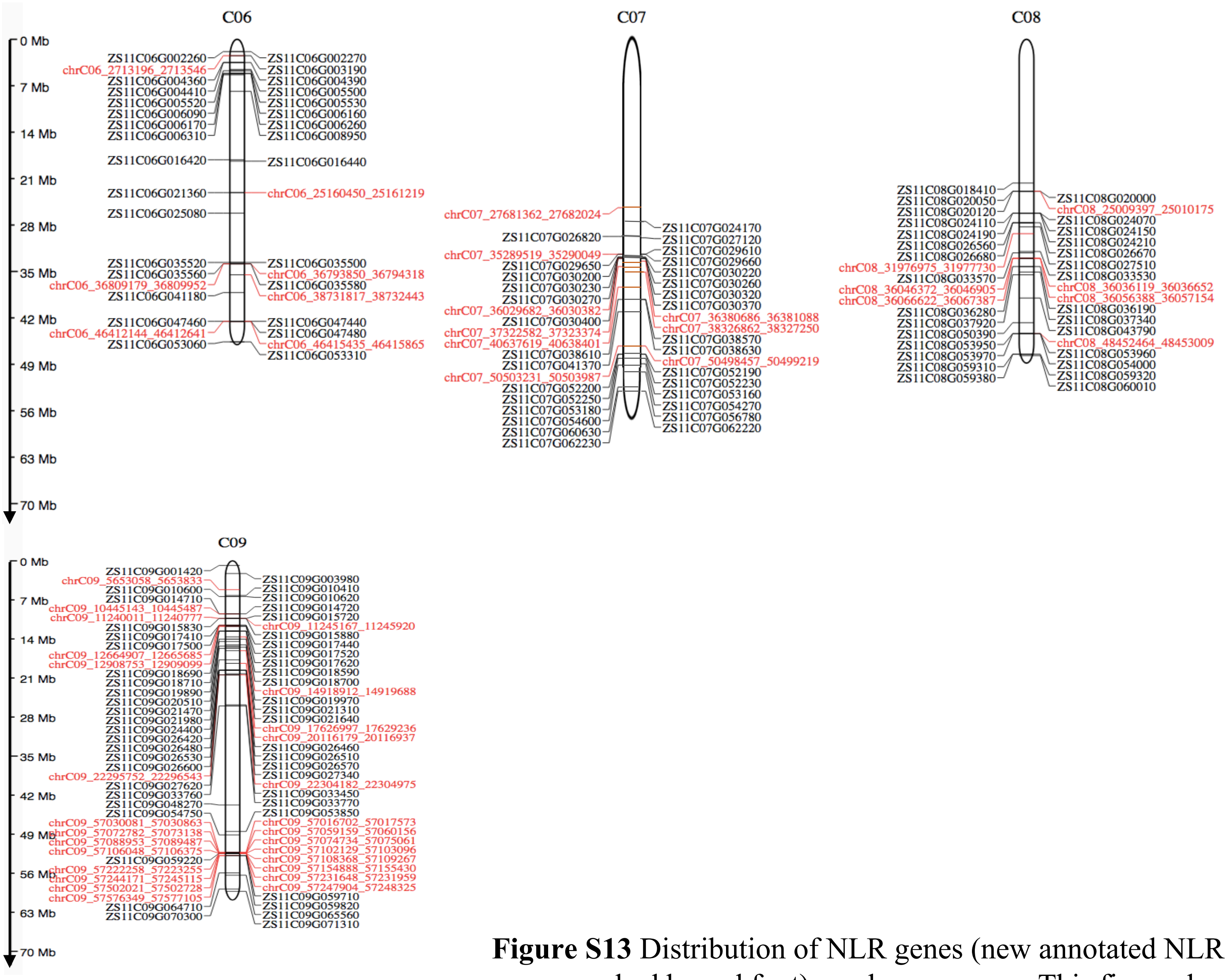

Figure S13 Distribution of NLR genes (new annotated NLR genes marked by red font) on chromosomes. This figure draw by online website MG2C v2.1 (http//mg2c.iask.in/mg2c_v2.1/).
