# Supplementary figures and images for: A high‐quality Brassica napus genome reveals expansion of transposable elements, subgenome evolution and disease resistance
Source: Plant Biotechnol J. 2020 Nov 20;19(3):615–30. doi: 10.1111/pbi.13493 (PMC7955885; doi:10.1111/pbi.13493)

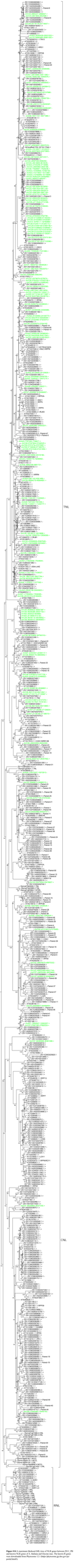

Supplement: Supplementary file 3 — Figure S14 A maximum likelihood (ML) tree of NLR genes between ZS11_PB and known NLR genes of A. thaliana and Glycine max. [file PBI-19-615-s003.pdf]
